# Fully automated CFD simulation system research based on design scheme tree

**DOI:** 10.1038/s41598-024-83582-2

**Published:** 2025-02-01

**Authors:** Zijun Liu

**Affiliations:** Hebei Petroleum University of Technology, Xueyuan Road, Chengde, 067000 Hebei People’s Republic of China

**Keywords:** Fully automation, CFD, Trend analysis, Engineering, Mathematics and computing

## Abstract

Compared with the remarkable achievements of computer-aided drug discovery systems for drug discovery, the role of computational fluid dynamics (CFD) in flow channel design requires further development. While CFD has undergone rapid evolution, the absence of integrated geometry and mesh processing hinders the potential development of advanced applications of this technology. To overcome this limitation, in this paper, the JIACFD toolset is presented, and a fully automated CFD simulation system is established. The simulation system is also constructed on a design scheme tree, which is more in accordance with engineering logic. The control parameter trend analysis method is introduced to select appropriate candidates from the design scheme tree. Additionally, the control parameter trend assumption, which is proven via the Spearman method, is proposed to improve the efficiency of the system. During the verification process for the study case, two independent control parameters exhibit correlating trends, and one control parameter converges when the number of meshes increases, indicating a lack of trend sensitivity. The design scheme tree and trend curve are subsequently utilized to effectively analyze the flow field characteristics of different schemes. Finally, the control parameter trend analysis method is employed to rank the design scheme tree and verify that the ranking of candidates is not dependent on the number of meshes. This paper investigates and verifies the presented system, method, and assumption and explores the possibility of an established system playing a more critical role in performance design work.

## Introduction

The design of the flow channel structure is intimately connected to the functionality of fluidic equipment. Consequently, numerous application fields must either innovate or optimize the structure. Rapidly evolving computer technology has led to extensive application of CFD-based design systems. In a design system, first, it is necessary to identify potential alternatives to determine the design parameters to be optimized. Second, it is essential to make performance predictions to obtain performance data under various design parameters. This prediction process can be combined with optimization algorithms to expand the search space for optimized design variables^1^. Ultimately, the data obtained can be used for decision-making purposes.

Performance prediction creates a relationship between design parameters and performance parameters. There are two main methods for prediction: the CFD simulation method and the surrogate modeling method (SMM). CFD simulation methods are based on physical models, which are complex and time-consuming to compute, but the data obtained are physically meaningful. In contrast, the SMM is an application of pure mathematical methods, which are characterized by simple and efficient calculation processes. However, the reliability of the data obtained is questionable. The CFD simulation method can be used alone, but the SMM must be used on the basis of existing data. Consequently, the SMM is often used in combination with CFD simulations. Hence, the CFD simulation method serves as a foundation for the system and is of paramount importance.

CFD-based analysis systems are being developed in numerous different fields, for example, for the preliminary development of an automated CFD-based analysis process for aircraft^2^, a web-based analysis process for automated CFD modeling of planing hulls^3^, an optimized building CFD model design process based on building information models^4^, and an automatic CFD simulation program for general purpose turbofan nacelles^5^.

All the above works involve automated processes; however, the implemented methods cannot break through the bottleneck^6^ in the traditional CFD simulation process; consequently, they are restricted by numerous constraints. Furthermore, it is regrettable that the intention and manner behind the use of automated processes remain unclear. Implementing automated processes as mere enhancements of CFD simulations is a misunderstanding of fully automated simulations.

The traditional workflow of CFD simulation encompasses three stages: preprocessing, flow field simulation, and postprocessing. In the traditional preprocessing phase, the geometric model is meshed to generate a mesh file. This process requires 3D modeling software and mesh generation software, which means that geometric model generation and mesh creation cannot be integrated together. This severely limits the flexibility of the simulation system to modify the geometric model. Therefore, many approaches have been developed to address this challenge. Among the numerous methodologies, these approaches can be classified into three distinct categories: development methods for specific fields, rebuilding methods and meshless methods.

The parametric design and rapid meshing (PADRAM) system is designed to facilitate the parametric modification of blade geometry and rapid generation of high-quality meshes^7^. This process combines several software programs to reconstruct surfaces from computed tomography (CT) image stacks and generate meshes^8^. The automatic mesh generation of cerebral arterial trees that can extract geometric features by distinguishing vascular skeletons has been studied via a parametric meshing procedure^9^.

These methods are constrained by the field of application, and the constructed systems are not universally applicable. The rebuilding method involves modifying the geometry or mesh through offset, scaling, or even twisting within a limited range on the basis of the existing geometric structure or mesh. These methods are more general and are widely employed in contemporary practice, mainly including free-form deformation and mesh morphing technology.

In free-form deformation, the matrix of control points encloses the entire geometry or specific region to be reshaped. Any movement of control points results in corresponding deformation of the actual surface^10,11^. Another prevalent method is mesh morphing technology, which morphs existing meshes according to geometric modifications to increase workflow efficiency^12^.

The meshless method has been rapidly developed^13,14^. Following the successful application of a meshless modelling technique to the simulation of transonic airfoils with automatically generated points, this method has been demonstrated to be the high flexibility of application^15^. Furthermore, a comparison of the simulation accuracy of meshless-based and mesh-based simulations has shown that meshless method has high engineering applications^16^. It is anticipated that meshless methods will significantly contribute to alleviating the limitations of contemporary CFD meshes.

The rebuilding and meshless methods allow straightforward and expeditious enlargement of geometrical structures to be simulated, thereby enhancing the flexibility of the simulation and analysis system. However, as the parameters to be modified become more extensive, the number of structures to be simulated also increases, resulting in a significant decline in system efficiency. This is particularly evident when guaranteeing the accuracy of the simulation results for each structure. In such instances, SMM can be employed to predict the corresponding performance, thereby improving the efficiency of the modification process.

Surrogate models, also referred to as approximation models, establish mathematical relationships between model geometric parameters and performance parameters, thereby enabling the prediction of performance, and widely used methods include the response surface method (RSM), kriging method and artificial neural network (ANN). The RSM establishes a regression equation between the response value and any parameter space point^17–19^. The kriging model is more suitable for cases requiring high accuracy and a large design space, and the generalized kriging model based on the Gaussian process, which consists of a deterministic linear function and a probabilistic error function, is widely used in combination with CFD simulations^20–24^. An ANN is a computational model inspired by the operation of nerve cells in living organisms. It is employed to address nonlinear systems or black-box models with complex internal representations^25,26^.

The SMM can be combined with optimization algorithms, such as traditional gradient algorithms^27^, genetic algorithms^26,28,29^, or simulated annealing algorithms^30^, to increase computational efficiency. If multiple criteria decision making (MCDM) is needed, then this method can be employed to assist decision makers in making optimal decisions. One convenient method for this purpose is the technique for order preference by similarity to an ideal solution (TOPSIS)^26,31^.

The systems above require significant investment of time and resources; however, the resulting outcomes are not particularly noteworthy. At the very least, they cannot be compared with the remarkable achievements of computer-aided drug discovery (CADD) systems^32^ in the pharmaceutical research field. Although the domains of industrial design and pharmaceutical research are distinct, the fundamental objective of harnessing computers to facilitate creative endeavors remains consistent.

For many years, research efforts in the CFD field have focused on improving simulation accuracy, such as the meshing of high-fidelity geometric structures, the development of various complex turbulence models, and multidisciplinary models^6^. Unfortunately, the bottleneck in the traditional preprocessing phase is not emphasized and solved, which ultimately leads to constraints on the role that CFD simulations can play in design work. The degree of innovation or optimization for a design problem, whether industrial, pharmaceutical, or otherwise, is contingent upon the breadth and number of study cases. Consequently, the greater the extent of coverage and the greater the number of cases simulated and analyzed are, the closer the results are to an ideal solution.

Accordingly, the establishment of a new CFD simulation system is more suited to the needs of the design field. In the process of establishing the simulation system, three key aspects must be addressed: the structure of the design scheme tree, the efficiency of the system, and the fully automated system.

1. The design scheme system is extensive, with numerous factors involved. It is essential to establish engineering logic and flexible design scheme tree systems.
2. According to the conventional simulation process, the dependence of the simulation results on the number of grids needs to be checked. As the number of simulated schemes increases and the verification process above is followed, the efficiency of the simulation system significantly decreases.
3. As the number of schemes to be simulated increases, the need to establish a fully automated simulation process becomes apparent.

This study establishes a design scheme tree in accordance with engineering logic. The independently developed JIACFD toolset is employed to achieve the integration of the geometric structure and mesh generation. This work enables the efficient generation of a mesh file pool. Subsequently, the flow field calculation program and postprocessing program are utilized to establish a fully automated CFD simulation process. The control parameter trend analysis method is employed to evaluate the design schemes. To improve application efficiency, a control parameter trend assumption is proposed. Finally, a specific case study is conducted to assess the feasibility and practicality of the system, method and assumption.

## System design philosophy

From a design perspective, comparing the predicted performance of as many schemes as possible is capable of improving design efficiency, whether at the early design phase or at the optimization phase. To achieve the above objectives, a large and significantly different sample space of flow channel structures should be established to obtain simulation data efficiently. However, specific modifications of the design scheme result in alterations to the flow field characteristics, which leads to the prediction method being more independent and more physically meaningful, and the CFD-based method is very appropriate. Therefore, it is necessary to develop a fully automated CFD simulation system to predict the performance of various design schemes to overcome the limitations and shortcomings mentioned in section “Introduction”. To construct such a system, the following factors should be considered:

1. Design scheme tree.
2. Control parameter trend analysis method.
3. Control parameter trend assumption.
4. Fully automated CFD simulation system.

### Flow channel design scheme tree

The variations in the design schemes are infinite, so the sample space needs to be determined first. According to the logical relationship, the design scheme tree (DST) is expanded from the initial scheme as the root of the tree. Following different engineering logic, design directions with significant differences can be formed, and then various design schemes can be established in each direction to form a scheme group taken as one branch of a tree. Different design schemes within the group are identified by changing local parameters and serve either as a root of a new branch or as a node on an existing branch.

Different design directions lead to significant design differences, which are manifested primarily in the definitions of feature components, including configuration components, layout, shape, detail dimensions, and even the number of inlets and outlets, resulting in a substantial parameter set. As the DST increases, the parameter set expands.

The original scheme $PO=\{po\}$ with geometric parameter set $C=\{c_j \mid j = 1,...,n\}$. The derivative scheme $PX=\{px_i \mid i=1,...,xm\}$ with all changed geometric parameters set $CX=\{cx_j \mid j = y1,...,yn\}$. The geometry matrix is created $GX=\{gx_{ij} \mid i=1,...,xm; j = y1,...,yn\}$ with the other parameters remaining still.

where $\{CX \subset C\}$ denotes that the modified parameters are selected from the original parameter set.

where $\{CX \cap C = \emptyset \}$ denotes that new parameters are extended from the original parameter set.

where $\{CX \cap C \ne \emptyset \ and \ CX \nsubseteq C \}$ denotes that the modified parameters are from the original set and that the others are new.

Then specific DST can be established. There are three branches with nodes:

$$PA=\{pa_i \mid i=1,...,am\}$$ with $$CA=\{ca_j \mid j = s1,...,sn\}$$,

$$PB=\{pb_i \mid i=1,...,bm\}$$ with $$CB=\{cb_j \mid j = t1,...,tn\}$$,

$PC=\{pc_i \mid i=1,...,cm\}$ with $CC=\{cc_j \mid j = n+1\}$ represents new parameters.

**Figure 1:**
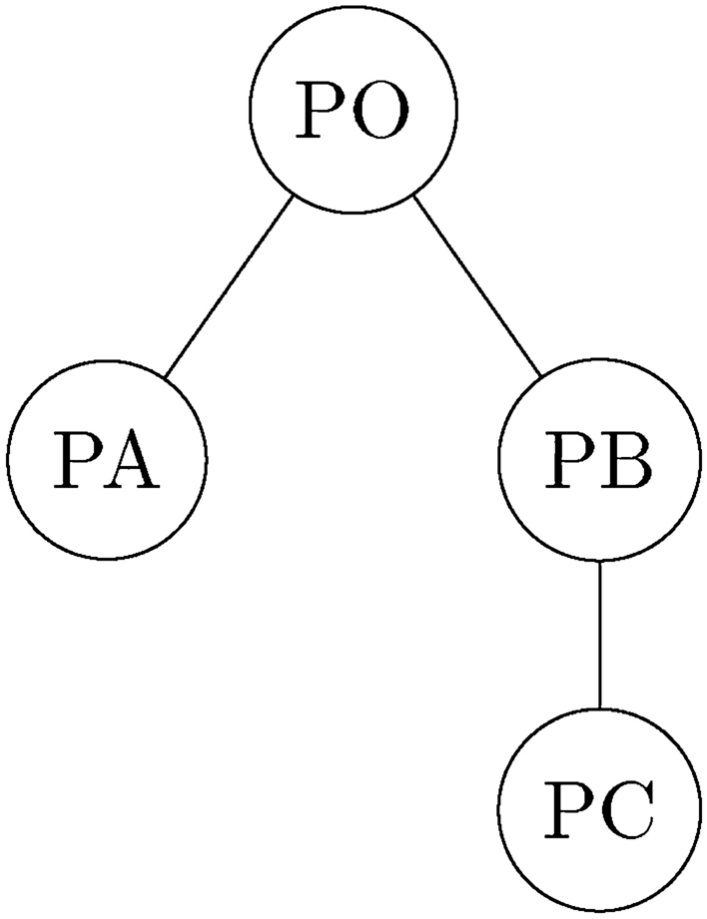
Design scheme tree structure.

The tree structure is illustrated in Fig. 1 and we obtain the DST matrix defined in Eq. 1.

1where, K denotes all the same parameter values, * denotes no change from the initial scheme.

### Control parameter trend analysis method

The control parameter trend analysis method selects one parameter as the control parameter of different schemes, the simulation results of which are plotted as a trend curve, according to which design schemes are studied to identify which design direction or scheme can significantly enhance performance. In this paper, the TOPSIS method in MCDM is selected as the trend analysis method for multiple control parameters.

The TOPSIS method is a frequently employed comprehensive evaluation method that fully utilizes the original data, and the results of the calculations demonstrate the performance differences between each scheme. The ideal best scheme set and the ideal worst scheme set are determined on the system data. Then the distance of each scheme from the corresponding ideal best scheme and ideal worst scheme is evaluated. The score of each scheme is calculated on the basis of these two distance values. Finally, the scores of all the schemes are ranked. A higher score correlates with a higher ranking, which indicates that the scheme performs better under this evaluation condition.

The performance of the flow channel reflects the combined influence of multiple control parameters. Turbulent flow is chaotic, and to eliminate the effect of transients, the time-averaged and spatial average calculation method is employed for controlling parameter sample values for data processing. Then, control parameter values for analysis can be obtained. Therefore, the control parameter trend mentioned in this and subsequent sections is based on control parameter values after data processing.

As for the design schemes(alternatives)$P=\{p_k \mid k = 1,..., n\}$ and the control parameters (criteria)$C=\{c_j \mid j = 1,...,m\}$, the performance matrix is created $X=\{x_{kj} \mid k=1,...,n; j = 1,...,m\}$ and $W=\{w_j \mid j = 1,...,m\}$ is the set of weights. The chosen scheme has the shortest distance from the determined positive ideal scheme $(A^+)$ and farthest distance from the determined negative ideal scheme $(A^-)$. The first step is to calculate the normalized performance matrix *Z* by Eq. 2.

2 $$\begin{aligned} z_{kj}(x)=\frac{x_{kj}}{\sqrt{\displaystyle \sum \nolimits _{k=1}^n {x_{kj}}^2}}, \quad k = 1,...,n; j = 1,...,m \end{aligned}$$

then to calculate the weighted normalized performance matrix by Eq. 3.

3 $$\begin{aligned} v_{kj}(x)=w_j z_{kj}(x), \quad k = 1,...,n; j = 1,...,m \end{aligned}$$

Next the positive ideal scheme and the negative ideal scheme are derived as:

4 $$\begin{aligned} A^+= & \Big \{ \Big ( \mathop {max}\limits _{k} v_{kj}(x)\mid j\in J_1 \Big ), \Big ( \mathop {min}\limits _{k} v_{kj}(x)\mid j\in J_2 \Big )\mid k = 1,...,n \Big \} \end{aligned}$$

5 $$\begin{aligned} A^-= & \Big \{ \Big ( \mathop {min}\limits _{k} v_{kj}(x)\mid j\in J_1 \Big ), \Big ( \mathop {max}\limits _{k} v_{kj}(x)\mid j\in J_2 \Big )\mid k = 1,...,n \Big \} \end{aligned}$$

where $J_1$ and $J_2$ are the positive impact and negative impact attributes, respectively.

Then, the distances from target scheme *k* to the best $v_j^+(x) \in A^+$ and worst $v_j^-(x) \in A^-$ should be measured.

6 $$\begin{aligned} d_k^+= & \sqrt{\sum \nolimits _{j=1}^m (v_{kj}(x) - v_j^+(x))^2 },\quad k = 1,...,n \end{aligned}$$

7 $$\begin{aligned} d_k^-= & \sqrt{\sum \nolimits _{j=1}^m (v_{kj}(x) - v_j^-(x))^2 },\quad k = 1,...,n \end{aligned}$$

The score can be derived as:

8 $$\begin{aligned} S_k = \frac{d_k^-}{d_k^+\ +d_k^- },\quad k = 1,...,n \end{aligned}$$

where $$S_k \in [0,1]$$ $$\forall k = 1,...,n$$.

Importantly, the process of obtaining effective sample values of control parameters is inevitably subject to error. Errors may arise from numerous sources, including mesh errors, errors associated with the simulation process, and errors due to the different sampling spaces needed. Traditional simulation requires high computational accuracy, but for the trend analysis method, as long as the trend of the parameter remains unchanged, it may not affect the ranking, so the accuracy requirement may be not high.

### Control parameter trend assumption (CPTA)

The design system is capable of expanding the range of design parameters and establishing a large simulation sample space. It is necessary to improve simulation efficiency and reduce simulation cost. In this paper, we propose the control parameter trend assumption to study the relationship between the trend curve and the number of meshes.

Control parameter trend assumption: For the design scheme tree, under the premise of whole simulation process error approximation, curves of the control parameter under different mesh series show an approximately consistent trend.

The term “Mesh Series” refers to the total set of mesh files formed by different mesh gaps. A list is formed with different mesh gaps, and one gap is selected for the mesh generation of all the schemes to obtain one subset of mesh files. These subsets are then collected together to form a total set. The number of meshes in the same subset may vary due to the differing geometric structures of various schemes.

The principal errors that may be encountered throughout the process include systematic errors, computational errors, mesh difference errors, and sampling errors. The systematic and computational errors can be controlled by using the same simulation platform. The mesh difference error refers to the mesh similarity of different design schemes; i.e., meshes of different schemes should be of uniform quality. The sampling error for different design schemes must be limited to different sampling areas to ensure that flow field information is obtained with the same characteristics.

Given the potential significant differences between the schemes in the DST, highly variable control parameter values are obtained from the simulation results, whose distributions deviate from a normal distribution. Therefore, the Spearman^33^ method is employed to test the above assumption.

Let $(X_1, X_2,...,X_n)$ and $(Y_1, Y_2,...,Y_n)$ be two samples of size *n*. $R_{X_i}$ denotes the rank of $X_i$ compared with the other values of the *X* sample, for $i = 1, 2,...,n$. Similarly, $R_{Y_i}$ denotes the rank of $Y_i$, for $i = 1, 2,...,n$.

The Spearman rank correlation coefficient, generally denoted by $\rho$, is defined as follows:

9 $$\begin{aligned} \rho = 1- \frac{6 \displaystyle \sum \nolimits _{i=1}^n d_i^2}{n(n^2-1)} \end{aligned}$$

where $$d_i=R_{X_i}-R_{Y_i}$$

### Fully automated CFD simulation process

It is imperative that full process automation be employed for efficient simulation of multiple design schemes. This paper presents a series of JIACFD tools that overcome the limitation of the preprocessing phase in the traditional simulation process. The flow channel geometry is templated, with geometric parameters defined in plain text files, and JIACFD tools are applied to read and generate mesh files, while the physical information of the flow channel is also defined. This process is more efficient in that it does not require the generation of any intermediate geometry file for transition.

**Figure 2:**
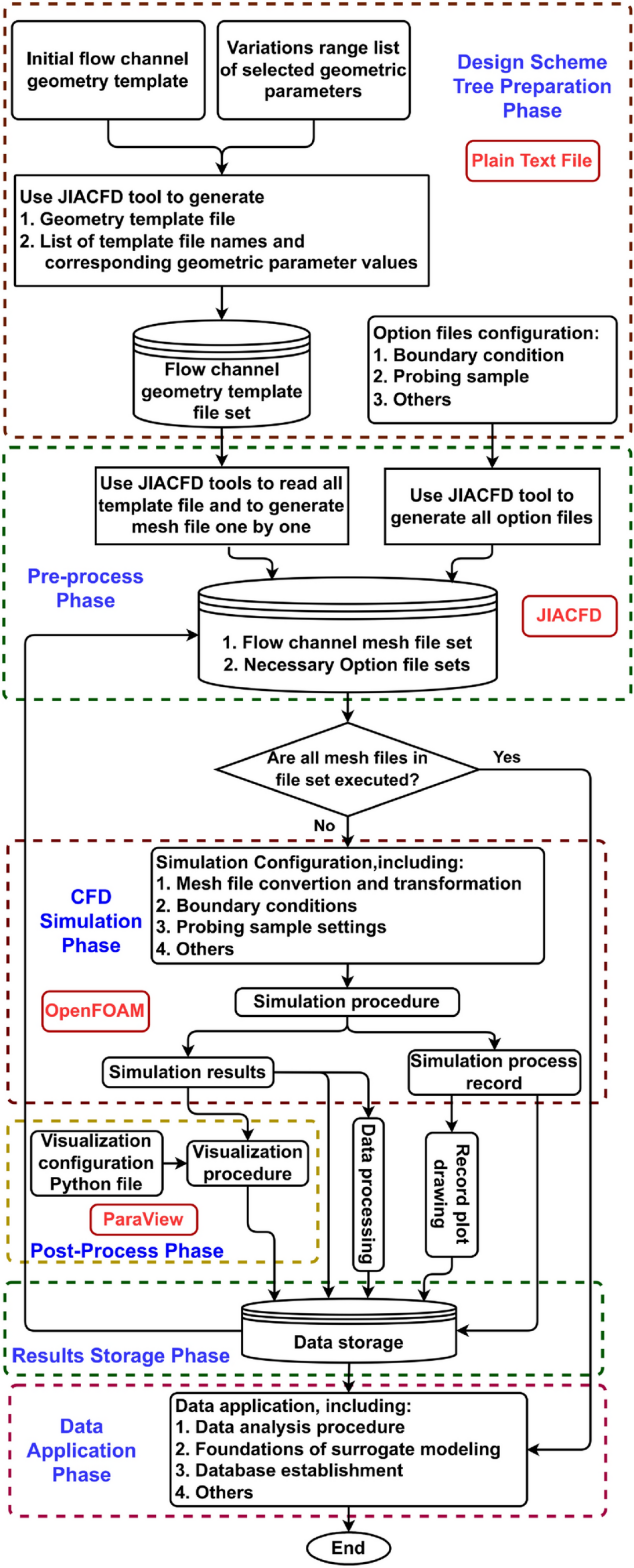
Fully automated CFD simulation system flow chart.

The fully automated simulation process is divided into six phases: design scheme tree preparation, preprocessing, CFD simulation, postprocessing, simulation result storage, and data application. The whole process is automated under the Ubuntu system via the Shell language. The specific process is illustrated in Fig. 2.

The preparation and data storage are new additional phases. The JIACFD toolset in the preparation phase and preprocessing phase comprises a group of C++ programs developed in this paper. The flow field simulation phase employs the well-known OpenFOAM^34^, which is an open-source computational fluid dynamics program, and standard solvers and models can be selected. ParaView, embedded with PvPython, is utilized to generate visualization results.

#### DST preparation

The DST is defined by establishing an initial scheme as a root and subsequently evolving various branches by modifying geometric structures that have the potential to optimize design on the basis of the root. The initial scheme template is first created and equipped with a list of variable variations. All scheme templates and the list of scheme name-variable correspondences are subsequently generated via JIACFD tools.

The construction of a DST is dynamic, and the tree structure grows as research progresses. The construction procedure is based on the engineers’ experience, reference to performance data, and predictive modeling. The establishment of a DST can be divided into three phases: (1)The initial stage is based on the engineer’s experience, and involves the selection of branches and the application of discrete parameters to define nodes on the basis of the initial scheme. (2)The growth phase defines nodes with continuous parameters on suitable branches, accumulates data, and explores mathematical models. (3)The intelligence phase entails the application of mathematical models to predict potential suitable branches and the continuous exploration of new schemes.

Appropriate branching is sufficient to produce significant alterations in the flow field, such as transitions from laminar to turbulent flow with constant initial fluid conditions. This approach allows for the effective utilization of the DST structure in response to substantial changes in the flow path.

Specific parameters can be selected according to both continuous and discrete approaches. The application of a continuous parameter set facilitates the establishment of a design scheme with a similar geometrical structure, and its simulation results exhibit a strong correlation, making it easy to establish a mathematical model. In contrast, discrete parameters have large numerical differences and the geometrical structures established are highly disparate, and their simulation results are relatively independent, which allows for the exploration of new branching directions.

It is necessary to create option files according to specific simulation requirements. These may include, for example, initial boundary conditions of velocities for simulation and configurations of coordinates for sampling points.

The format of all files in this phase is a plain text file, which is more flexible for various applications.

#### Preprocessing

**Figure 3:**
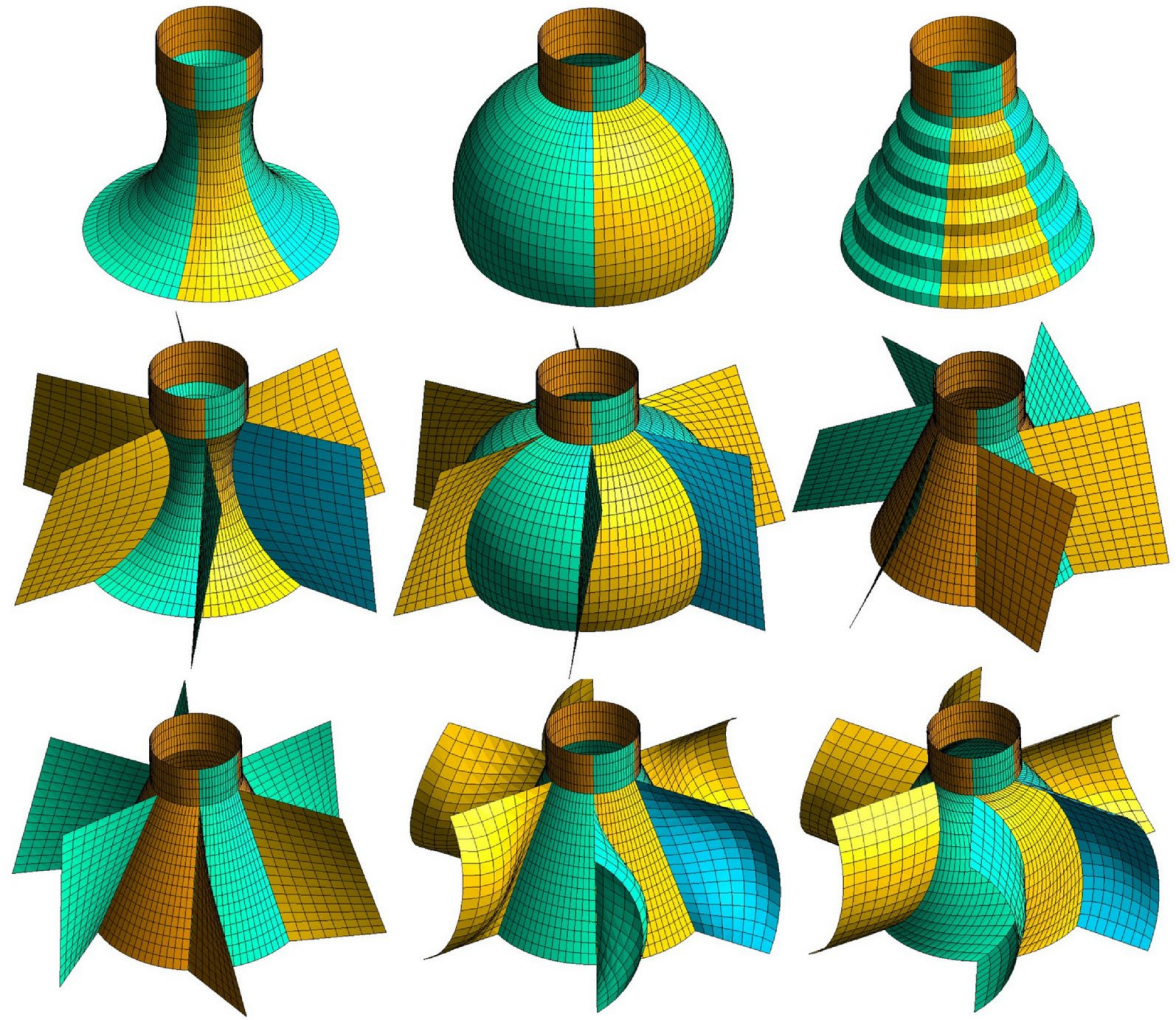
Different embedded curve applications.

**Figure 4:**
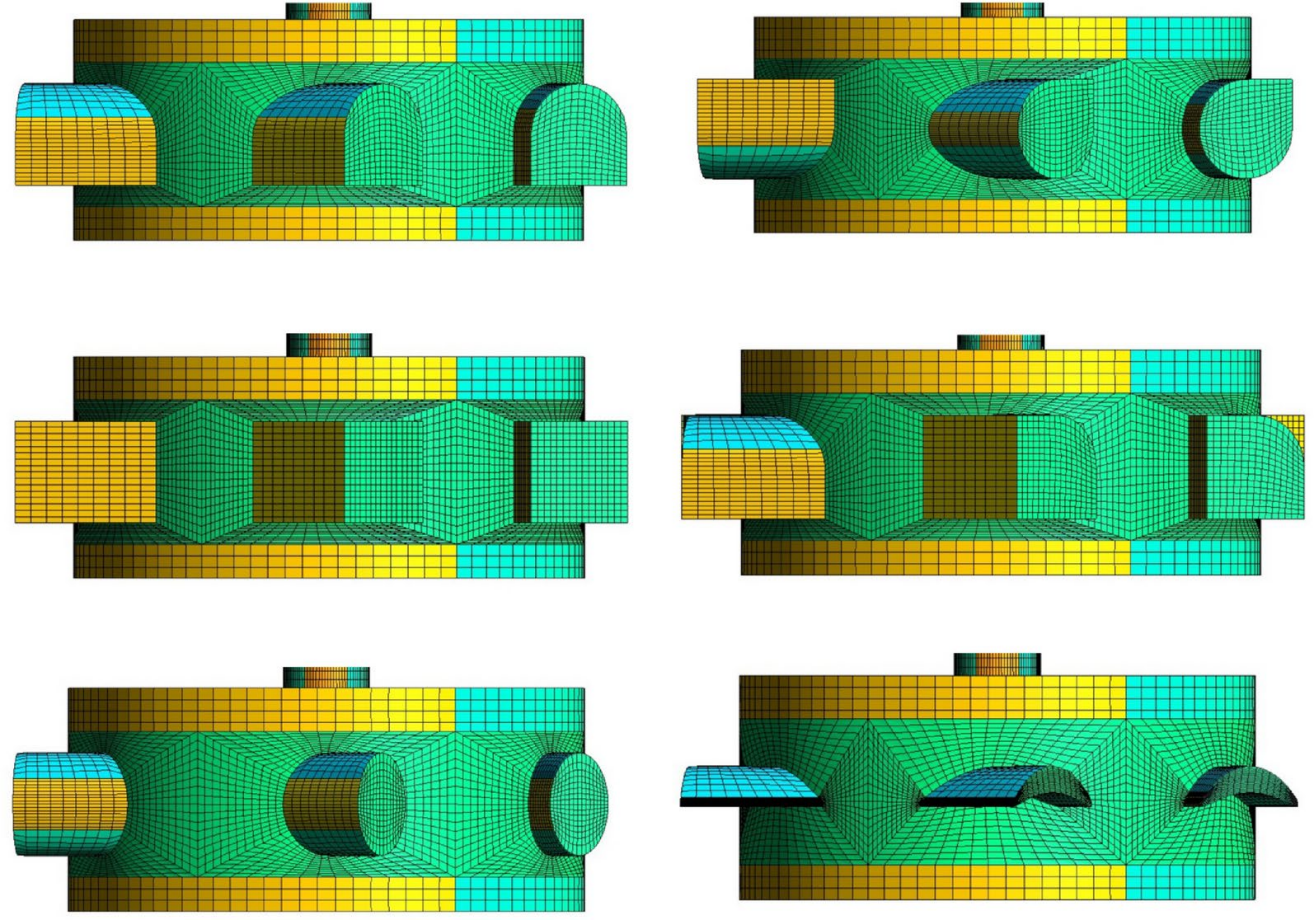
Different embedded shape applications.

In this phase of work, JIACFD tools are employed to read in geometric text templates and option configurations to generate a mesh file set and corresponding option file set for enabling realization of the preprocessing flow of this simulation system. The JIACFD tools integrate fully parametric geometric modeling with structural meshing algorithms from the Gmsh library^35^. The fully parametric technique definitively states that any shape can be constructed through a set of parameters. Simple geometries are constructed from basic parameters such as length, width, height, radius, etc. Complex objects require parameters that are obtained through formula calculations or the importing of standard geometric data. All parameters can be set as absolute or relative values or a combination of values. A set of parameters is taken as input to select the frame points that can be divided into multi-block. Based on the frame points set, the geometry is generated by using the OpenCASCADE graphics algorithm (the generated geometry can be exported separately if needed). The geometry can be used as subsequent meshing. The primary alterations implemented by JIACFD for the flow channel encompass the following four elements:

1. The internal parts can be defined by internal surfaces to affect internal flow, as illustrated in Fig. 3.
2. The component shape can be configured according to a specified curve. The initial step is to create the desired unit-size shape curve or profile. This is then embedded into the geometric structure by positioning, scaling, rotating, and so forth to achieve the desired shape, as shown in Fig. 4.
3. The component layout refers to the relative relationships among components, including their relative positions and directions.
4. The component detailed dimensions are those that pertain to size, length, and other local characteristics of a component under the condition that the shape has been determined.

The fully parametric geometric modeling approach allows for flexibility in making major changes to the geometry, as well as necessary local changes, while the use of text templates effectively enhances the flexibility of the modelling approach.

In this work, we utilize a structured hexahedral mesh^36^ generated by transfinite interpolation method^37–39^, which is ordered and can effectively regulate the discrepancy in mesh quality between different design schemes. The transfinite interpolation method is the most representative algebraic mesh generation method, and its most prominent feature is that the distribution of mesh points and the orthogonality of the mesh at the boundary can be readily controlled. The major advantages of the transfinite interpolation mesh method are the ease of application, the low computational cost, and the capacity to regulate the shape and density of the mesh in a relatively straightforward manner. The most significant advantage of this approach is that it can reduce the time required to generate a mesh in comparison to other hexahedral mesh generation methods^40^. The mesh gap is used as a control factor to refine the mesh and regulate the number of meshes. The mesh file types include msh, cgns, su2, unv, and others.

The JIACFD toolset is written in pure C++ and employs the OpenCASCADE graphics library API, the Eigen library, and the Gmsh library API^35^ in addition to standard libraries such as iostream and math. The final compilation is carried out with the GNU g++ tool.

The integrating geometry and mesh generation is essential for building an automated system, and this integration effectively addresses the bottlenecks mentioned previously. It has a higher degree of extensibility and applicability than the traditional preprocessing methods and specific mesh generation methods mentioned in section “Introduction”. Therefore the JIACFD toolset is the best choice for establishing a fully automated CFD simulation system.

#### CFD simulation

The set of mesh files created in the previous step serves as the file pool, and the loop operation continuously runs until all mesh files in the pool have completed simulation calculations.

The OpenFOAM library is employed for flow field computations. Concurrently with the looping of mesh files, corresponding option files are configured from the option file pool. Prior to flow field simulation, mesh transformation is performed. After simulation, sample point data acquisition is completed automatically, and the data corresponding to the coordinates must be extracted in accordance with the definition in the configuration file. Finally, the results of the simulation are stored for subsequent postprocessing. Additionally, the process record is utilized for plotting the curve and then stored.

The utilization of an open-source calculation program facilitates a reduction in hardware and software costs when a two-level parallel computing system is established^1^. The first level is the macro-operation, which requires the construction of a set of workstations and the even distribution of cases to be simulated on each workstation. The second level is the micro-operation, which applies the MPI library for the thread-level administration of single-case simulation on the foundation of multi-core CPUs on each workstation.

#### Postprocessing

The objective of postprocessing of the system is automatic generation of visualization files. This is achieved through the use of modules provided by PvPython embedded in ParaView, which allows Python language to be applied to write configuration files for specific purposes, such as streamlines and contours. Finally, PvPython executes configuration files to generate visualization files.

#### Data processing and storage

In addition to the data referenced in section “CFD simulation”, the results of the visualization presented in the previous section must also be stored.

In this work, the simulation data is stored in folders, which are then numbered according to the scheme index number. The data can be extracted and applied using a management program written in shell language.

In this phase, all control parameter sample values must be data processed to calculate their temporal and spatial averages to obtain their analysis values. First, a time series is defined with different specified time points for sampling. Second, the spatial average of the sampled values for each time point is calculated. Finally, the average spatial average value of each time point is calculated as a temporal average to obtain control parameter analysis values.

#### Data application

The final phase is data application, which can be used for data management, data analysis and decision-making purposes. It can be integrated into SMM, and it can also be used for database creation. Furthermore, this phase can be separated from the automated system as needed to facilitate flexible application of various data analysis methods and software.

## Case study

**Figure 5:**
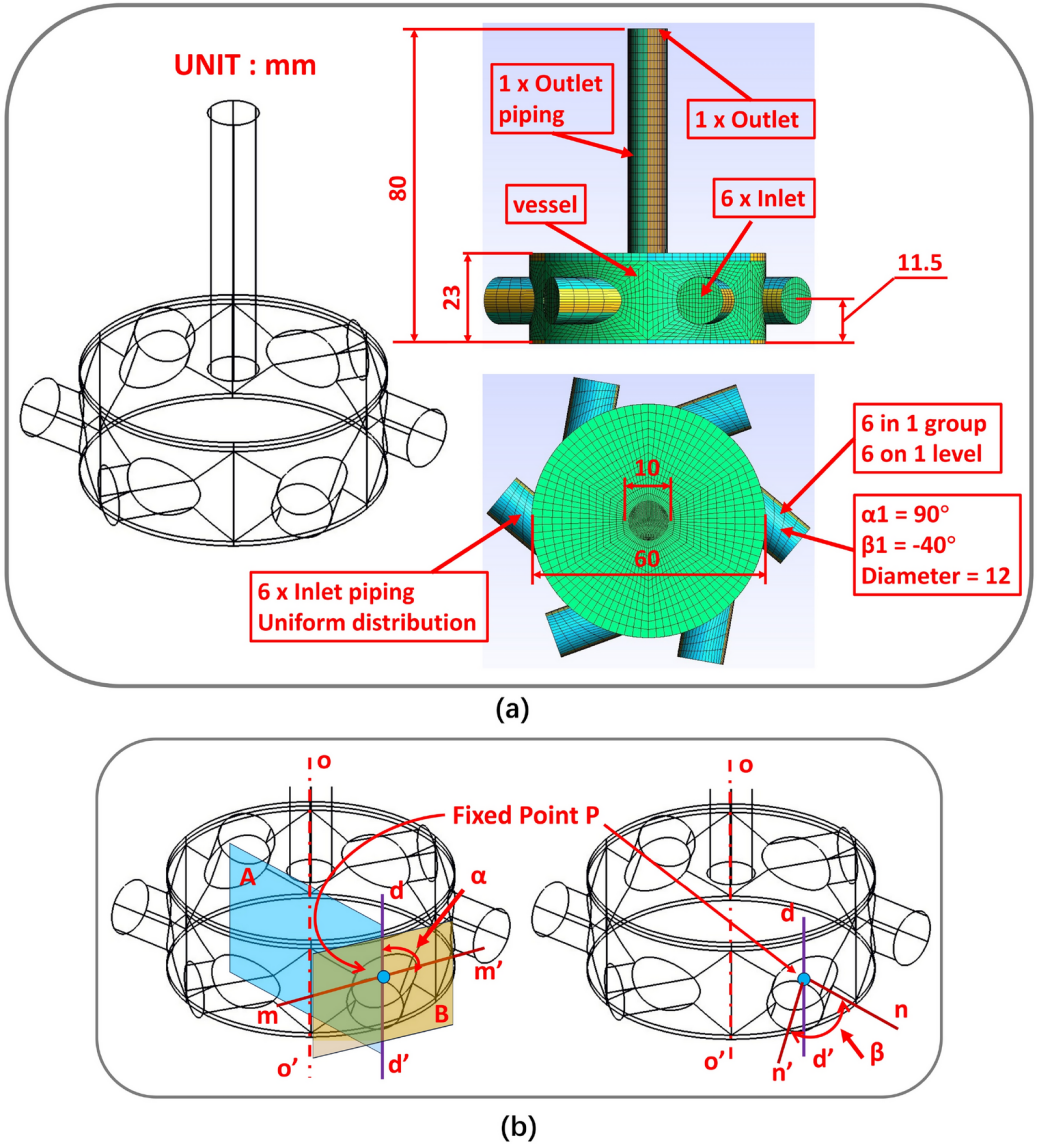
Initial scheme detail. (**a**) Detailed parameters of the initial scheme structure. (**b**) Inlet piping rotation operation settings.

In this work, a vessel flow channel with six inlets and one outlet is constructed to test the feasibility of the simulation system. The initial scheme structure is shown in Fig. 5a, which is highly expandable. A DST can be constructed by changing the inlet and outlet dimensions, arranging the inlet piping, and increasing the internal components. When the inlet direction is changed, the boundary conditions need to be changed as well. Once appropriate boundary conditions have been assigned to various schemes, the feasibility of the simulation process established in section “System design philosophy” can be thoroughly evaluated.

The fluid enters the vessel through each of the six inlets and exits through the outlet pipe, where it flows and mixes within the vessel. The piping arrangement is altered primarily by rotating inlet pipes and adjusting their relative positions.

The rotation operation is based on set of angles [$\alpha$, $\beta$]. Here $\alpha$ is the angle between line $dd'$ and line $mm'$, and line $mm'$ is the fixed base that is perpendicular to axis $oo'$ in tangent surface *B* over fixed point *P* which is the intersection point of the pipe axis and the vessel surface, and line $dd'$ is the intersection line of surface *A* and the vessel surface that passes through fixed point *P*. The angle of rotation of the normal *n* of the vessel surface over the fixed point *P* with line $dd'$ as the axis is denoted by $\beta$. The axis of pipeline $n'$ is obtained after rotation as illustrated in Fig. 5b. If six inlet pipes are divided into *x* groups, then an angle configuration$\{[\alpha 1, \beta 1],......, [\alpha x, \beta x]\}$ is required to meet the inlet pipe configuration requirements.

The simulation settings are determined, including the use of water as the fluid medium at $20 ^{\circ } C$, a velocity of 5*m*/*s* for all inlets, a simulation time of 0.2*s*, and boundary conditions. The stable RANS-komegaSST model, which is included in the OpenFOAM library as a standard model, is selected as turbulence calculation model.

Four control parameters, namely velocity magnitude, differential kinematic pressure, turbulence intensity and Péclet number^41^, are selected as the control parameters for evaluating the design scheme. Please refer to Eqs. 10, 11, 12 and 13 listed below.

The magnitude velocity *magU* [*m*/*s*] is defined as:

10 $$\begin{aligned} \vec {U}= & U_x\vec {i}+U_y \vec {j}+U_z \vec {k}\nonumber \\ magU= & \sqrt{U_x^2+U_y^2+U_z^2} \end{aligned}$$

The differential kinematic pressure $\Delta P$ between sample point and the discharge, $[m^2/s^2]$

11 $$\begin{aligned} \Delta P= & P_{sample} - P_{discharge}\nonumber \\ \text {where:} \nonumber \\ & {{P_{discharge} = 0 \, m^2/s^2}} \end{aligned}$$

The turbulence intensity field *I* is defined as:

12 $$\begin{aligned} I= & \frac{\sqrt{\frac{2}{3}k}}{U} \end{aligned}$$

where, k is turbulent velocity fluctuations, [m/s], U is local velocity magnitude, [m/s].

The Péclet number *Pe* is defined as:

13 $$\begin{aligned} Pe= & \frac{Vh}{\kappa } \end{aligned}$$

where, V is characteristic velocity, [m/s], h is length scales, [m], ${\kappa }$ is scalar diffusivity, [m$^2$/s].

The working platforms are as follows: All the programs are executed on the Ubuntu-20.04 platform, which uses a single-processor Intel Core CPU i5-8500 at a frequency of 3.00 GHz. The JIACFD tool is employed for preprocessing, and nonparallel simulations are performed via OpenFOAM-10.0 and the standard solver pimpleFoam. Postprocessing is conducted via Paraview-5.10.0 and Python-3.6.9, resulting in the generation of visualization results. Finally, SPSSAU-24.0^42^ is used for data analysis.

### DST establishment

**Figure 6:**
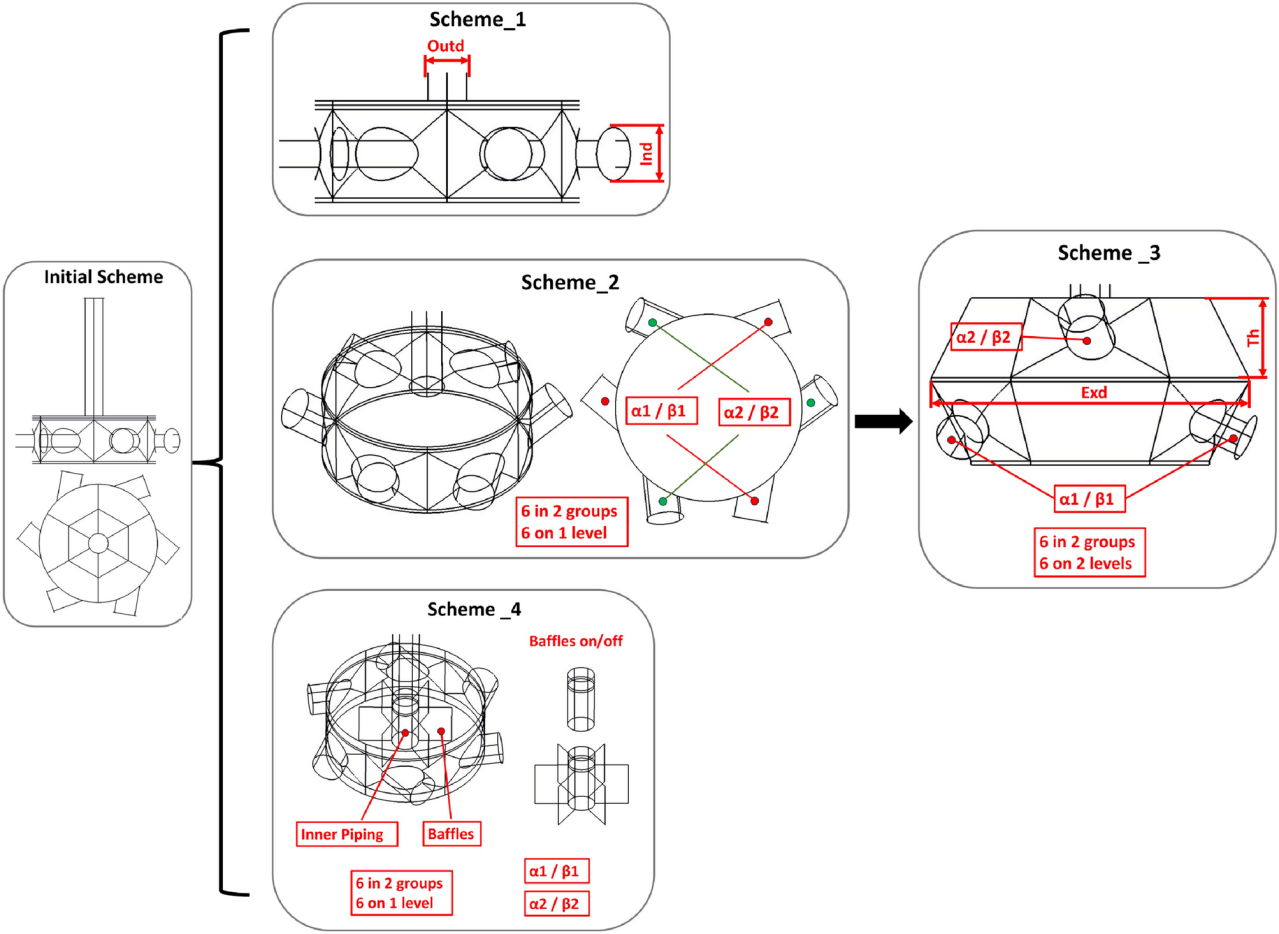
Design scheme tree.

This paper establishes the DST, as shown in Fig. 6. It creates four branches, the first of which is modified by the sizes of the inlet and outlet pipes. The diameter of the outlet pipe is designated *Outd*, whereas the diameter of the inlet pipe is designated *Ind*. The second branch adjusts the inlet pipe arrangement. The inlet pipes are in the same layer, with six pipes divided into two groups, and the sizes of the inlet and outlet pipes are fixed. The control angles ($\alpha$1, $\beta$1) and ($\alpha$2, $\beta$2) are used to determine arrangement, which is divided into two types: two groups with the same rotational arrangement and two groups with the opposite arrangement. The third branch involves the distribution of inlet pipes in two layers. The height *Th* of the upper layer of the vessel is increased such that it is equal to the height of the lower layer. Additionally, the walls of the two layers are tilted by increasing the diameter *Exd* of the transition section in the middle of the upper and lower layers. The six inlet pipes are divided into two groups, and the angles of the inlet pipes [$\alpha$1, $\beta$1] and [$\alpha$2, $\beta$2] are adjusted. The fourth branch increases internal elements, namely internal piping or internal piping with baffles, with a gap$=4mm$ from the vessel bottom to the internal piping bottom. This branch divides six inlet pipes into two groups, control angles ($\alpha$1, $\beta$1) and ($\alpha$2, $\beta$2), and increases the height of the upper level of vessel.

**Figure 7:**
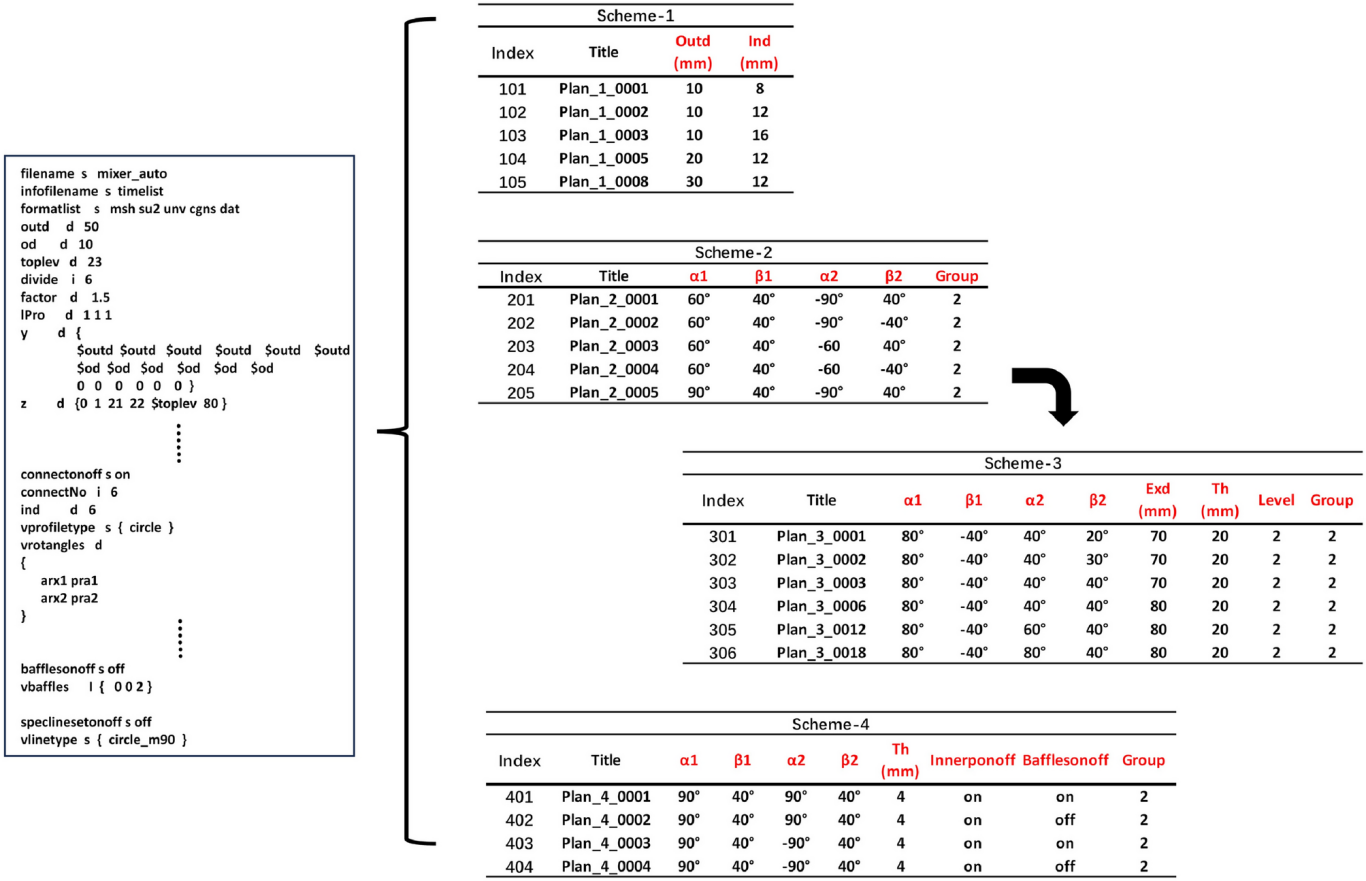
Design parameter tree.

**Figure 8:**
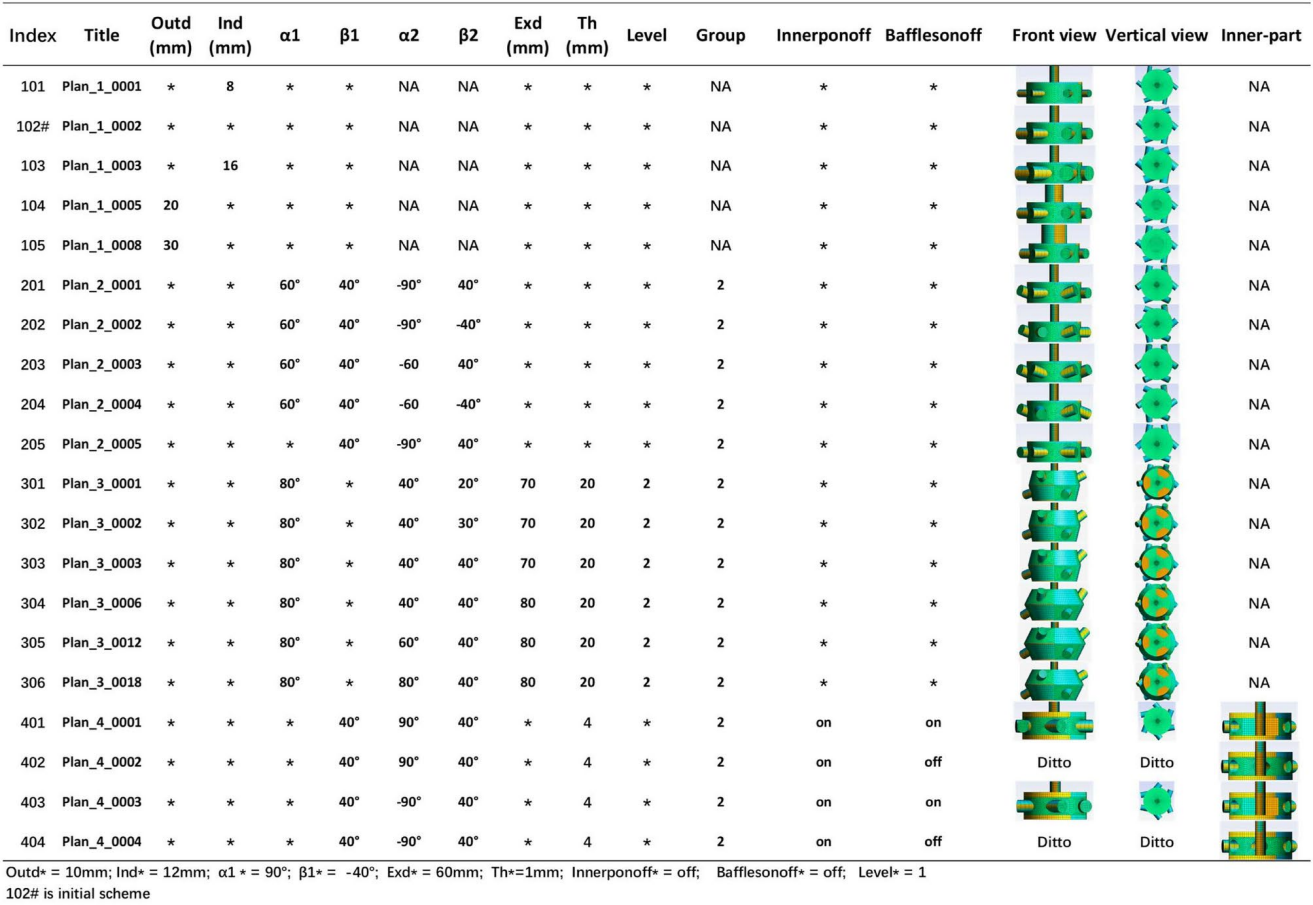
Design scheme matrix.

The corresponding parameter values are presented in Fig. 6 and Fig. 7. Finally, the design scheme matrix is constructed in Fig. 8. The matrix lists the design scheme index number, the corresponding name of the generated scheme, and the values of all the parameters that have been altered, as well as the corresponding profile. According to the definition of DST, an infinite number of schemes can be generated. In this work, some typical schemes are selected as sample cases for verifying the automated simulation process and assumptions, so the index numbers of the scheme names are not continuous.

### Option files

The option files that must be configured for this case include inlet velocity boundary conditions and sampling point configurations.

**Figure 9:**
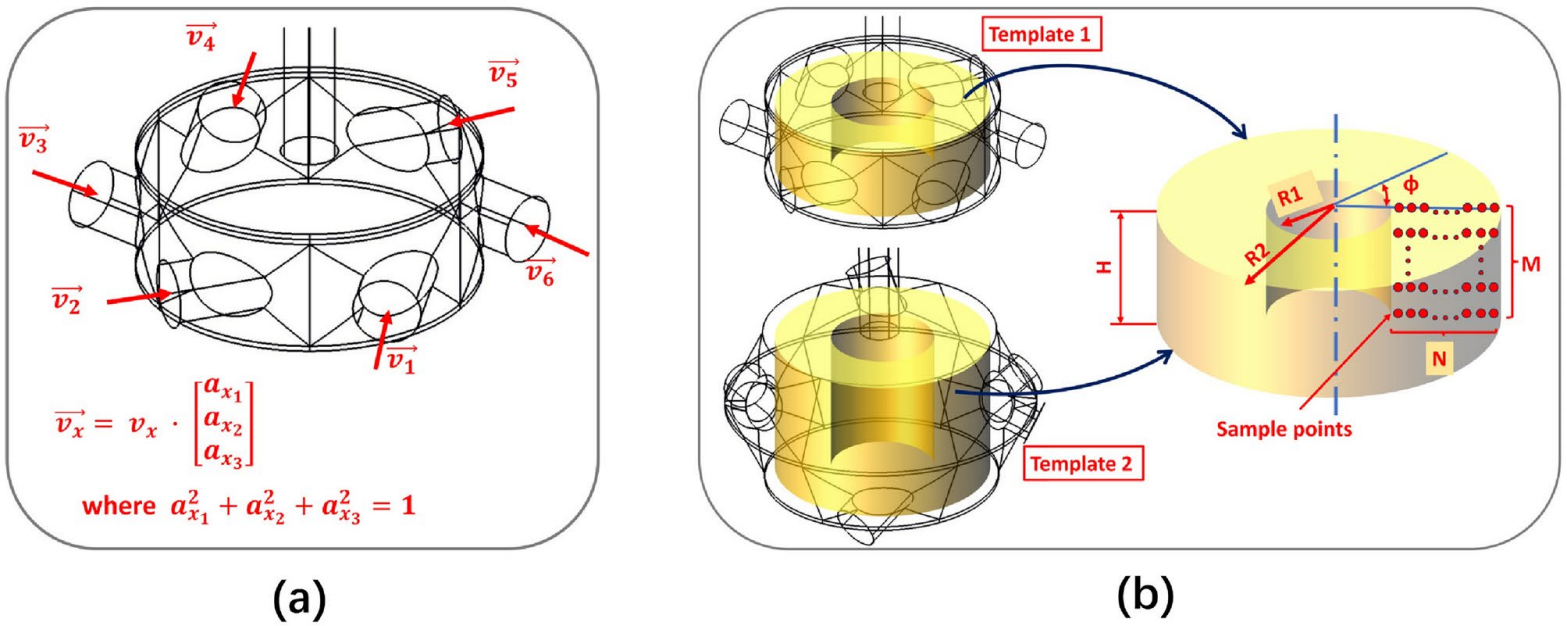
Option files. (**a**) Inlet velocity boundary condition. (**b**) Probing the sample configuration template.

The velocity boundary conditions must be set for simulation. Since the six inlet directions are different, it is necessary to calculate velocity vectors for each inlet separately when establishing initial conditions for inlet velocities. To improve automation efficiency, for each scheme, the corresponding initial velocity file is generated at the same time as the mesh file is generated, as illustrated in Fig. 9a.

According to the DST established in section “DST establishment”, the flow channel space of Schemes 3 and 4 varies considerably. The sampling space needs to be adjusted to avoid the influence of special regions, as shown in Fig. 9b. Scheme 4 is configured with internal piping, and the internal flow field changes significantly. Therefore, the sample space excludes this region, and the annular region is used as sample space. The inlet pipe of Scheme 3 is arranged in two layers, which increases the height of the vessel, and consequently, the sample space. Scheme 3 uses template 2 as the sample space, whereas schemes 1, 2, and 4 use template 1. Two templates are employed within the same number of sampling points, see Table 1 for detailed settings.

**Table 1:** Settings for the templates.

	H(mm)	$\hbox {G}^{[1]}$ (mm)	R1 (mm)	R2 (mm)	$\phi$	M	N	$\hbox {TN}^{[2]}$
Template1	18	2	6	26	$40^{\circ }$	10	11	990
Template2	38	2	6	26	$40^{\circ }$	10	11	990

$^{[1]}$ G is a gap from the sample region bottom to the vessel bottom.

$^{[2]}$ TN is total number of the sample points

### Fully automated process verification

**Figure 10:**
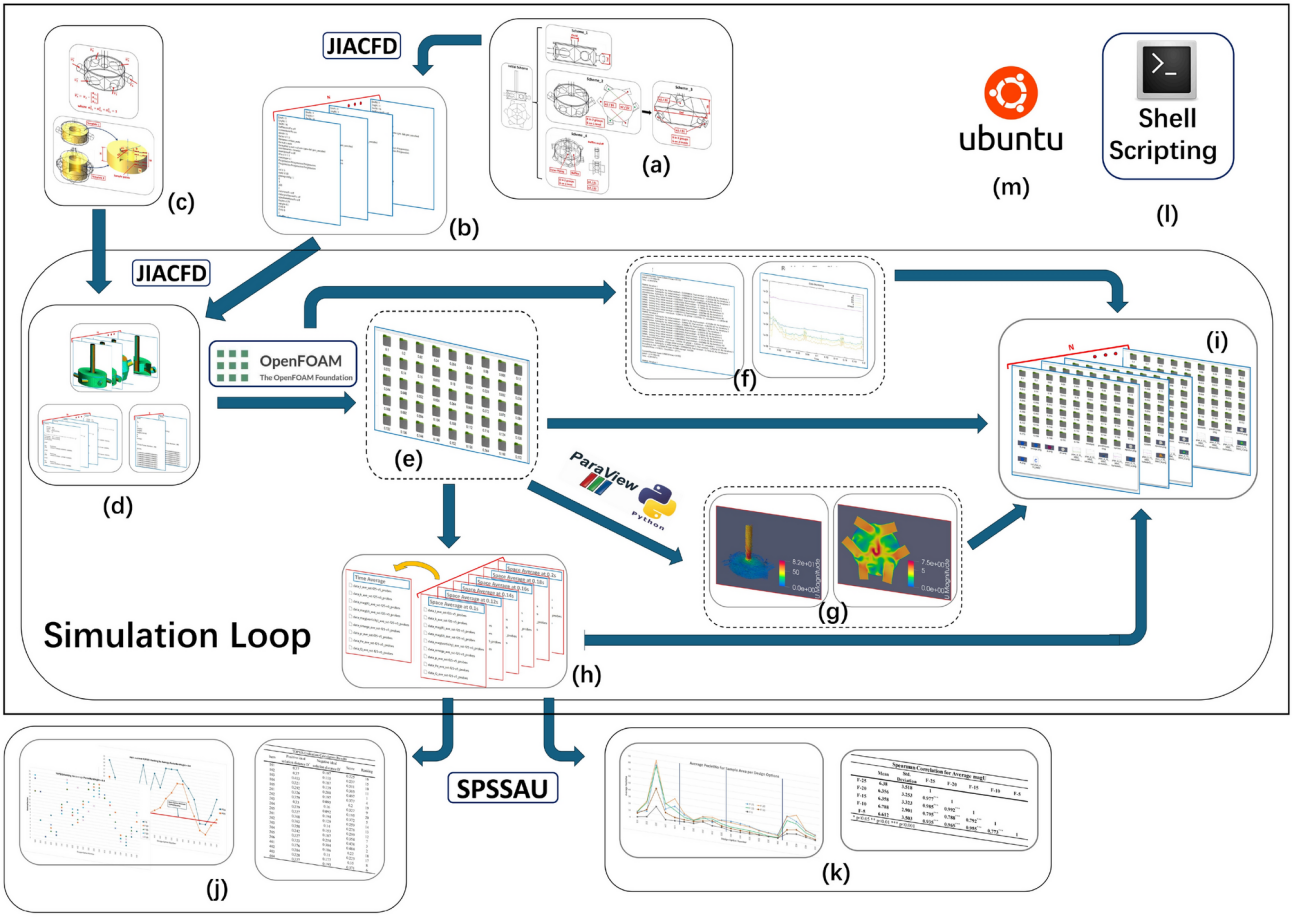
Fully automated simulation process. (**a**) Design scheme tree. (**b**) Flow channel geometry template file set. (**c**) Inlet velocity boundary condition and probing sample configuration template. (**d**) Flow channel mesh file set, inlet velocity boundary condition file set and probing sample configuration file set. (**e**) Simulation results. (**f**) Simulation process record and plot drawings. (**g**) Visualization results. (**h**) Data processing for time-spatial average calculation. (**i**) Data storage. (**j**) TOPSIS evaluation calculation results and plot drawing. (**k**) Control parameter trend plot drawing and Spearman correlation calculation. (**l**) Shell scripting used for auto procedure. (**m**) Ubuntu platform.

The fully automated CFD process described in section “Fully automated CFD simulation process” allows us to visualize the flow of this case as shown in Fig. 10.

The mesh series is [2.5, 2.0, 1.5, 1.0, 0.5] with units of millimeters, corresponding to series marks ${F-25, F-20, F-15, F-10, F-5}$. The corresponding mesh numbers, mesh generation times, and simulation times are shown in Table 2. As the mesh gap decreases, the number of meshes increases, whereas the mesh generation time remains relatively constant, approximately 160*s* are needed to generate a mesh file. The simulation time increases as the number of meshes increases. The JIACFD toolset is feasible and efficient for preprocessing to generate DST.

**Table 2:** Mesh information and time costs.

Index	Title	F-25			F-20			F-15			F-10			F-5		
		PPT$^{[1]}$ (sec)	SMT$^{[2]}$ (sec)	HN$^{[3]}$	PPT$^{[1]}$ (sec)	SMT$^{[2]}$ (sec)	HN$^{[3]}$	PPT$^{[1}$] (sec)	SMT$^{[2]}$ (sec)	HN$^{[3]}$	PPT$^{[1]}$ (sec)	SMT$^{[2]}$ (sec)	HN$^{[3]}$	PPT$^{[1]}$ (sec)	SMT$^{[2]}$ (sec)	HN$^{[3]}$
101	Plan_1_0001	153	5487	42492	151	10542	60966	152	9941	99387	155	28204	188226	162	60324	577422
102	Plan_1_0002	155	20778	42492	151	17625	60966	151	36075	99387	157	102237	188226	158	173376	577422
103	Plan_1_0003	154	39684	42492	151	56573	60966	151	76046	99387	158	189686	188226	157	308616	577422
104	Plan_1_0005	148	1571	38844	151	1948	52614	149	2682	87579	154	5555	162882	155	23940	501282
105	Plan_1_0008	158	528	35196	157	664	48438	155	1148	75771	160	2408	145986	160	15213	425142
201	Plan_2_0001	151	27854	42492	151	44641	60966	155	41535	99387	155	114757	188226	159	267580	577422
202	Plan_2_0002	151	24602	42492	151	31703	60966	155	38153	99387	158	102509	188226	161	216342	577422
203	Plan_2_0003	154	22963	42492	151	38535	60966	155	55607	99387	155	124502	188226	162	261326	577422
204	Plan_2_0004	155	24815	42492	153	23285	60966	153	40862	99387	157	101778	188226	159	201942	577422
205	Plan_2_0005	151	33749	42492	153	40274	60966	154	50338	99387	153	122404	188226	158	175108	577422
301	Plan_3_0001	151	15520	46137	152	22684	68256	149	39807	116667	151	103359	226431	162	259342	736587
302	Plan_3_0002	157	16006	46137	149	23106	68256	153	38370	116667	154	86485	226431	163	267973	736587
303	Plan_3_0003	149	14926	46137	151	28933	68256	153	44370	116667	155	97581	226431	159	258337	736587
304	Plan_3_0006	152	17145	46137	153	24644	68256	153	36801	116667	155	71198	226431	162	321352	736587
305	Plan_3_0012	149	17405	46137	151	28247	68256	151	39655	116667	156	85241	226431	163	313996	736587
306	Plan_3_0018	149	16652	46137	149	28550	68256	149	41058	116667	151	86139	226431	160	321857	736587
401	Plan_4_0001	151	24164	41976	152	51019	59877	152	72142	93789	153	209695	178884	159	276020	558144
402	Plan_4_0002	151	22826	41976	154	41987	59877	155	53823	93789	157	126882	178884	163	266046	558144
403	Plan_4_0003	154	29663	41976	152	46639	59877	151	67240	93789	152	195695	178884	158	248598	558144
404	Plan_4_0004	153	36964	41976	150	53718	59877	156	60934	93789	157	183462	178884	158	261675	558144

$^{[1]}$ PPT is Preprocessing Time.

$^{[2]}$ SMT is Simulation Time.

$^{[3]}$ HN is Hexahedra Number

### Assumption verification

The assumption presented in section “Control parameter trend assumption (CPTA)” is verified by the ${F-25, F-20, F-15, F-10, F-5}$ series of simulation results. The time–space average values of four control parameters $\Delta P$, *magU*, *Pe* and *I* are employed for verification.

**Figure 11:**
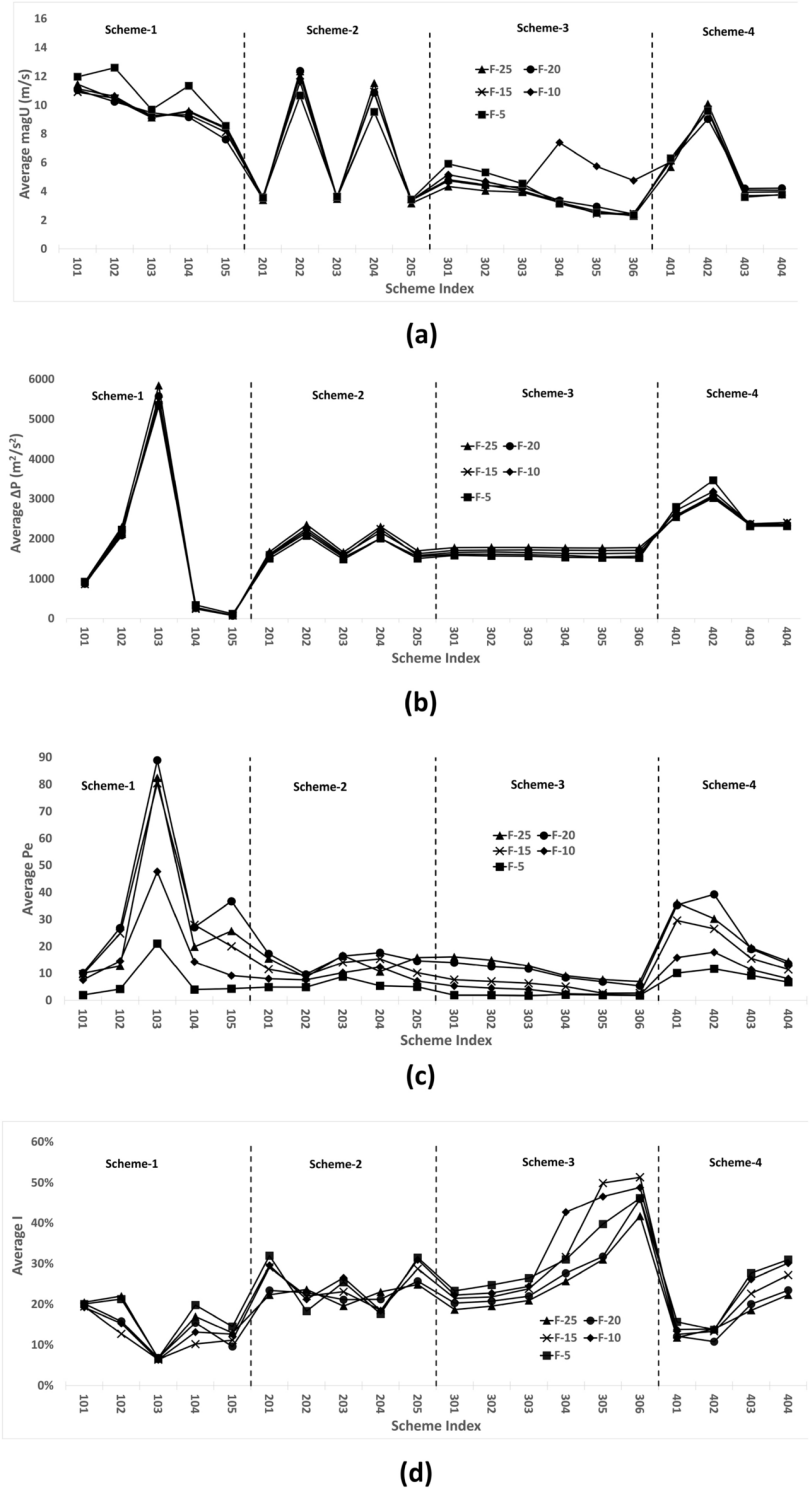
Design scheme tree trend curve comparison for the [F-5, F-10, F-15, F-20, F-25] series for (**a**) magU. (**b**) $\Delta P$. (**c**) Pe. and (**d**) I.

Spearman data analysis revealed a significant positive correlation, as illustrated in Tables 3, 4, 5 and 6 which are corresponding to Fig.11a–c and d respectively. Therefore, the trend assumption is verified for the four selected control parameters in this case.

**Table 3:** Spearman correlation for the average magU.

	Mean	$\hbox {SD}^{[1]}$	F-25	F-20	F-15	F-10	F-5
F-25	6.38	3.518	1				
F-20	6.356	3.253	$$0.977^{***}$$	1			
F-15	6.358	3.323	$$0.985^{***}$$	$$0.992^{***}$$	1		
F-10	6.788	2.901	$$0.795^{***}$$	$$0.788^{***}$$	$$0.792^{***}$$	1	
F-5	6.612	3.503	$$0.935^{***}$$	$$0.965^{***}$$	$$0.955^{***}$$	$$0.773^{***}$$	1

$$^{*}p<0.05.\quad ^{**}p<0.01.\quad ^{***}p<0.001.$$

$^{[1]}$ SD is Std. Deviation

**Table 4:** Spearman correlation for the average $\Delta P$.

	Mean	SD$^{[1]}$	F-25	F-20	F-15	F-10	F-5
F-25	2006.259	1148.058	1				
F-20	1942.52	1111.247	$$0.988^{***}$$	1			
F-15	1918.906	1105.044	$$0.989^{***}$$	$$0.985^{***}$$	1		
F-10	1890.673	1091.274	$$0.967^{***}$$	$$0.973^{***}$$	$$0.982^{***}$$	1	
F-5	1890.677	1111.032	$$0.974^{***}$$	$$0.980^{***}$$	$$0.988^{***}$$	$$0.989^{***}$$	1

$$^{*}p<0.05.\quad ^{**}p<0.01.\quad ^{***}p<0.001.$$

$^{[1]}$ SD is Std. Deviation

**Table 5:** Spearman correlation for the average Pe.

	Mean	SD$^{[1]}$	F-25	F-20	F-15	F-10	F-5
F-25	19.319	16.646	1				
F-20	21.603	18.729	$$0.889^{***}$$	1			
F-15	16.916	17.1	$$0.814^{***}$$	$$0.950^{***}$$	1		
F-10	10.62	9.878	$$0.737^{***}$$	$$0.908^{***}$$	$$0.973^{***}$$	1	
F-5	5.695	4.727	$$0.642^{**}$$	$$0.699^{***}$$	$$0.762^{***}$$	$$0.806^{***}$$	1

$$^{*}p<0.05.\quad ^{**}p<0.01.\quad ^{***}p<0.001.$$

$^[1]$ SD is Std. Deviation

**Table 6:** Spearman correlation for the average I.

	Mean	$\hbox {SD}^{[1]}$	F-25	F-20	F-15	F-10	F-5
F-25	0.209	0.073	1				
F-20	0.209	0.086	$$0.935^{***}$$	1			
F-15	0.229	0.117	$$0.818^{***}$$	$$0.932^{***}$$	1		
F-10	0.243	0.115	$$0.800^{***}$$	$$0.920^{***}$$	$$0.983^{***}$$	1	
F-5	0.244	0.093	$$0.741^{***}$$	$$0.854^{***}$$	$$0.925^{***}$$	$$0.949^{***}$$	1

$$^{*}p<0.05.\quad ^{**}p<0.01.\quad ^{***}p<0.001.$$

$^{[1]}$ SD is Std. Deviation

As illustrated in Fig. 11, numerous schemes exhibit variations in control parameters across different numbers of meshes, which is unacceptable for single-scheme simulations, because the criterion of mesh independence is not followed. Nevertheless, it does not affect the trend results of multiple-scheme simulations, which can be utilized for trend analysis.

### Trend correlation of different control parameters

**Figure 12:**
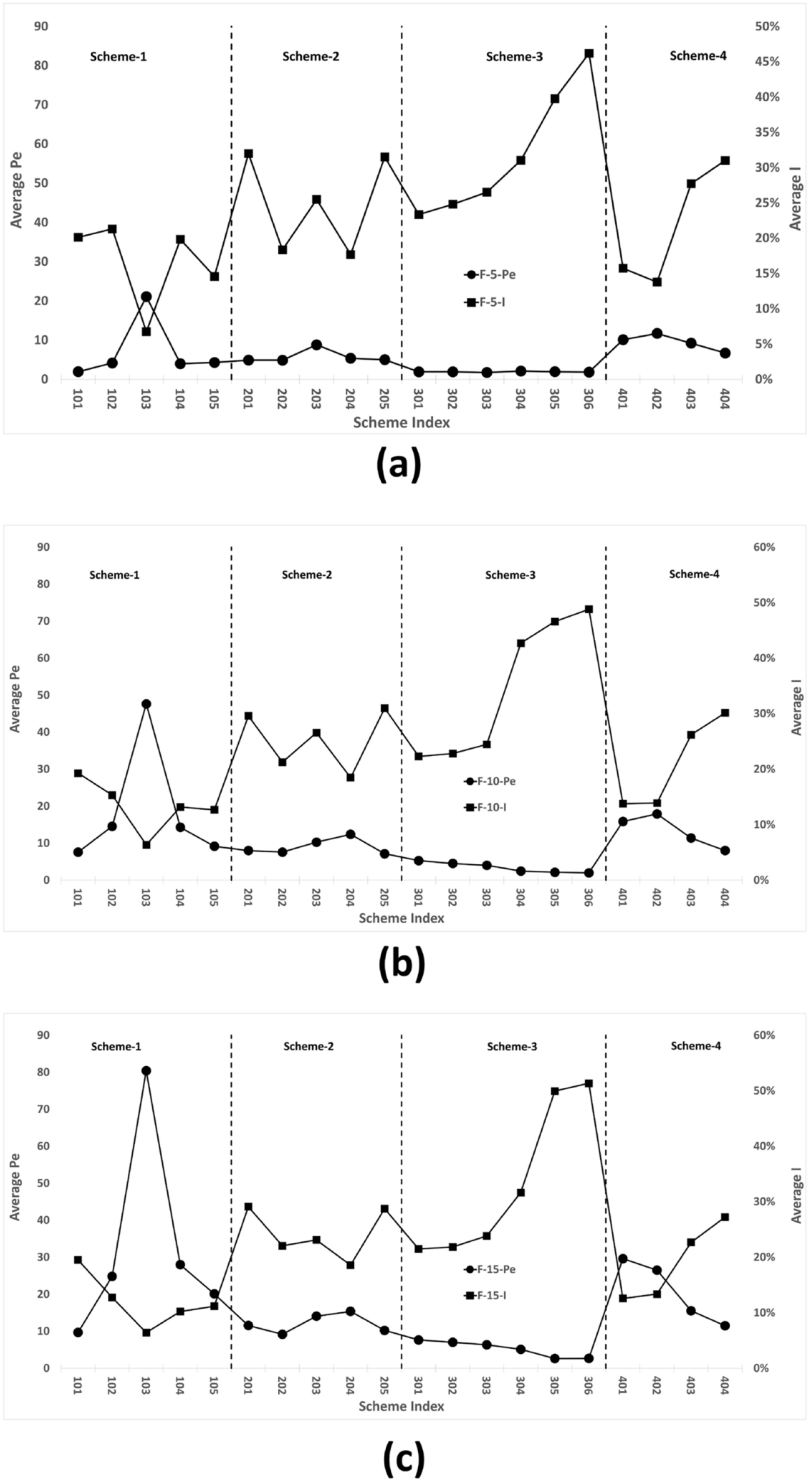

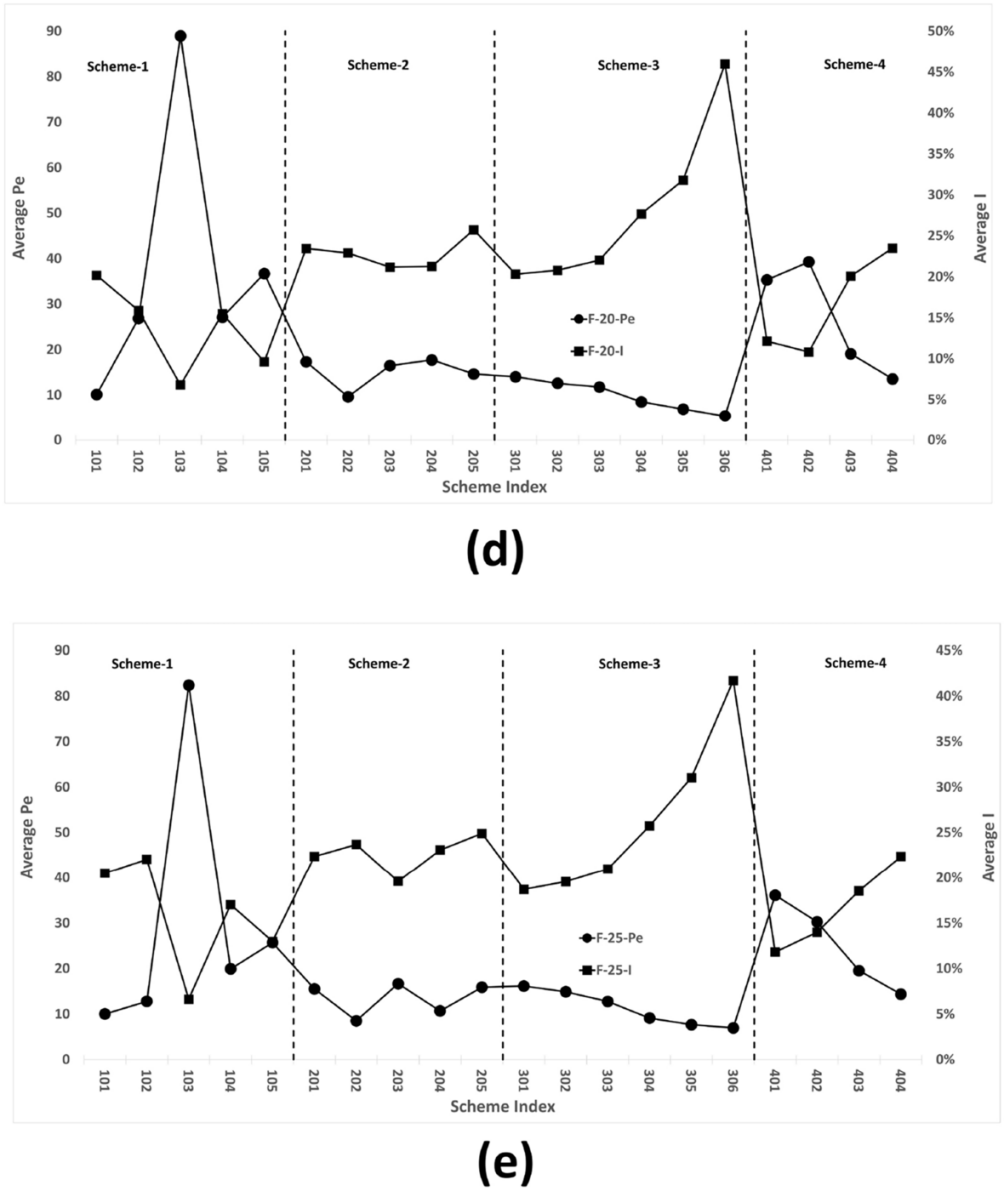
Design scheme tree trend comparison between Pe and I for (**a**) F-5, (**b**) F-10, (**c**) F-15, (**d**) F-20, and (**e**) F-25.

In the previous section, the trends of two items, *Pe* and *I*, are verified. The opposite trend is apparent, which is demonstrated in Fig. 12. Table 7 shows the results of Spearman data analysis. This analysis reveals that the trends of *Pe* and *I* in all series have highly significant negative correlations see Table 7.

**Figure 13:**
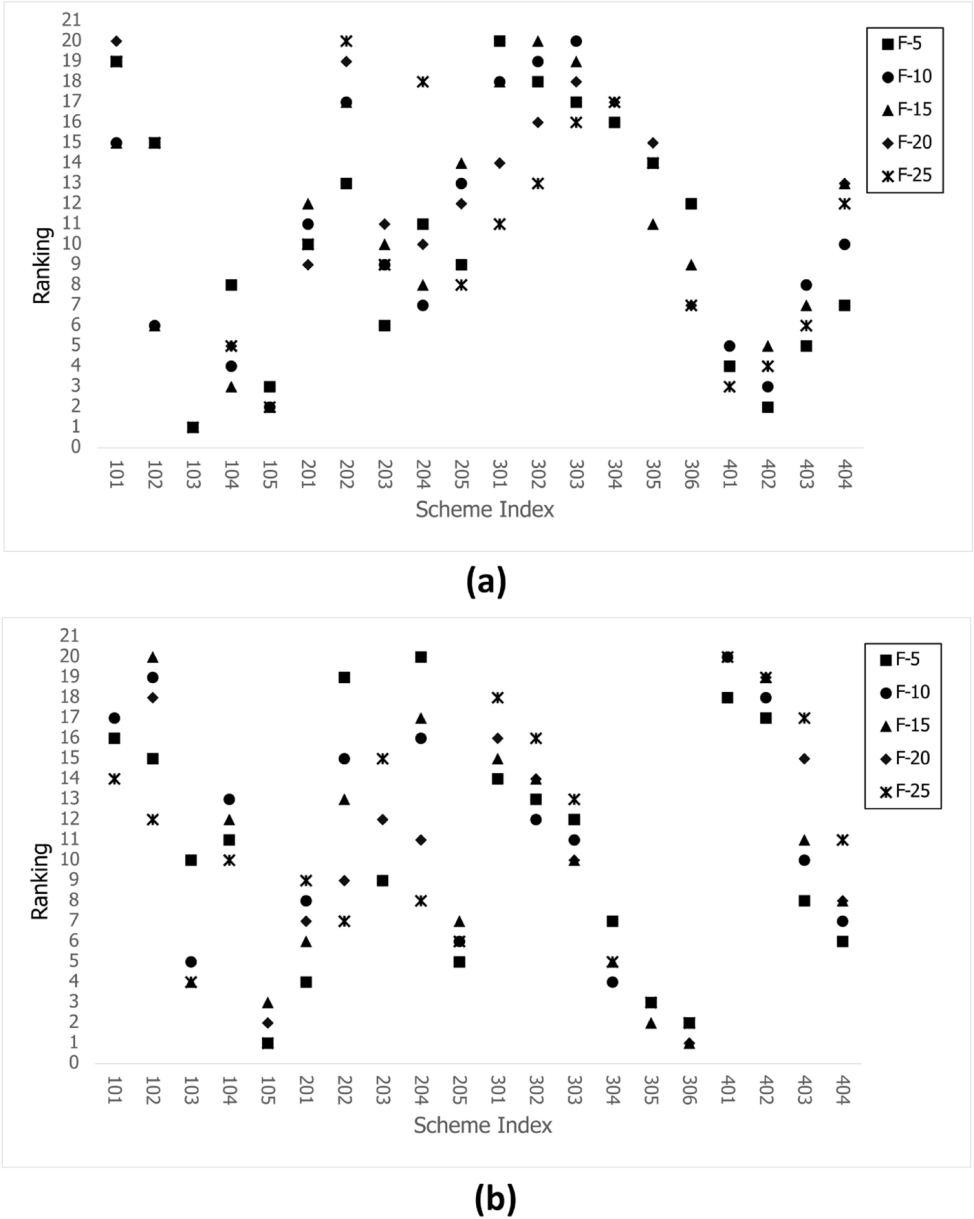
TOPSIS rankings for the [F-5, F-10, F-15, F-20, F-25] series for (**a**) Pe weight=0.8 and (**b**) I weight=0.8.

**Figure 14:**
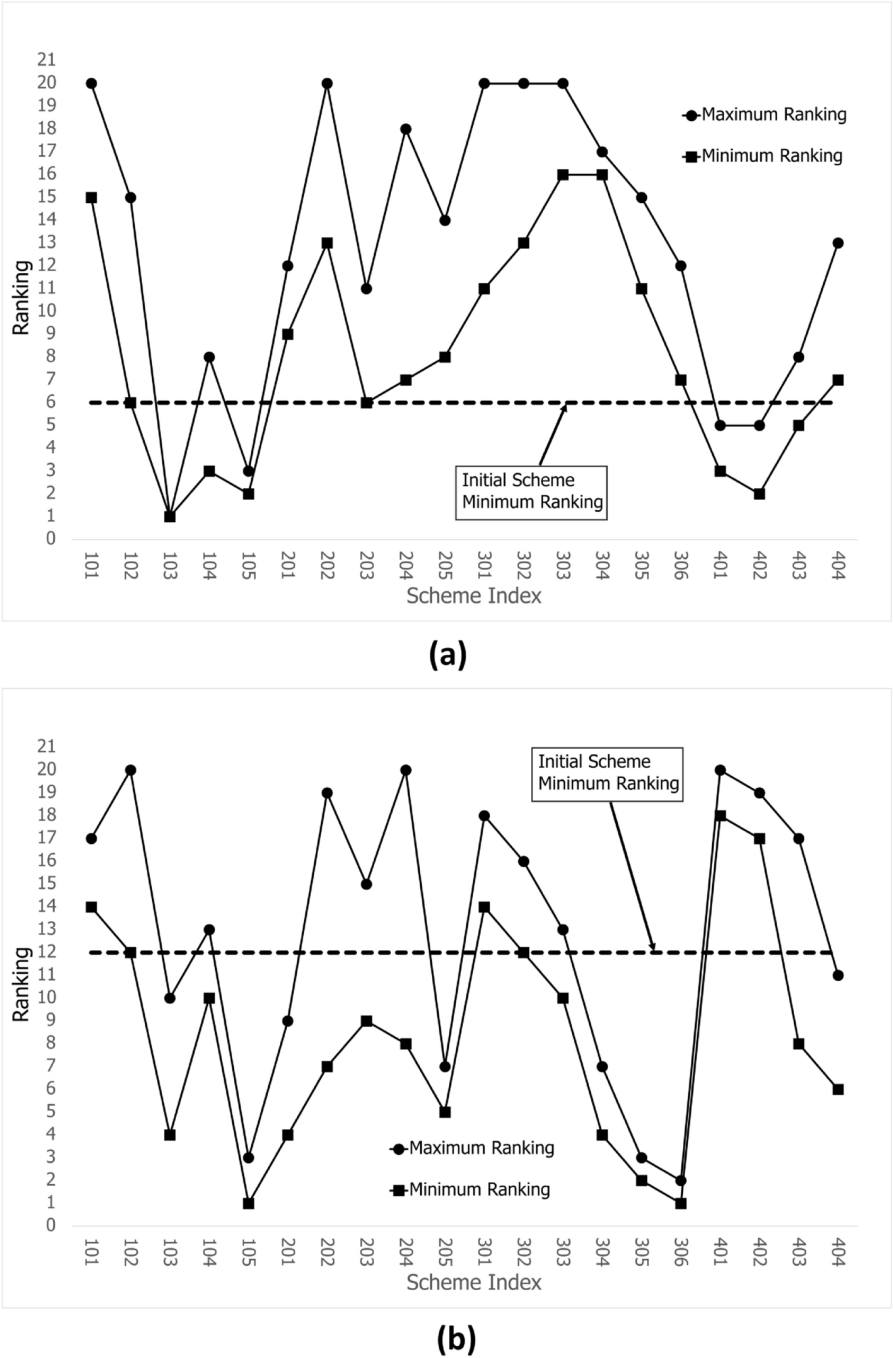
Maximum and minimum TOPSIS rankings from the [F-5, F-10, F-15, F-20, F-25] series for (**a**) Pe weight=0.8 and (**b**) I weight=0.8.

From Eqs. 12 and 13, *Pe* and *I* are two independent parameters. In conjunction with the design scheme figures from section “DST establishment”, two distinct cases can be distinguished. The first case pertains to instances where the *Pe* value is elevated, and the corresponding schemes exhibit a rotational arrangement in the same direction as that of the initial scheme, particularly the Scheme-1 area. In the second case, the *I* value is high, and the corresponding inlet pipe arrangement is divided into two groups of opposite arrangements. The main schemes include 205, Scheme-3 area and 403, 404 (Figs. 13, 14).

**Table 7:** Spearman correlation for Pe and I.

	F-25	F-20	F-15	F-10	F-5
Coefficient	$$-0.889^{***}$$	$$-0.848^{***}$$	$$-0.806^{***}$$	$$-0.767^{***}$$	$$-0.450^{*}$$

$$^{*}p<0.05.\quad ^{**}p<0.01.\quad ^{***}p<0.001$$

**Figure 15:**
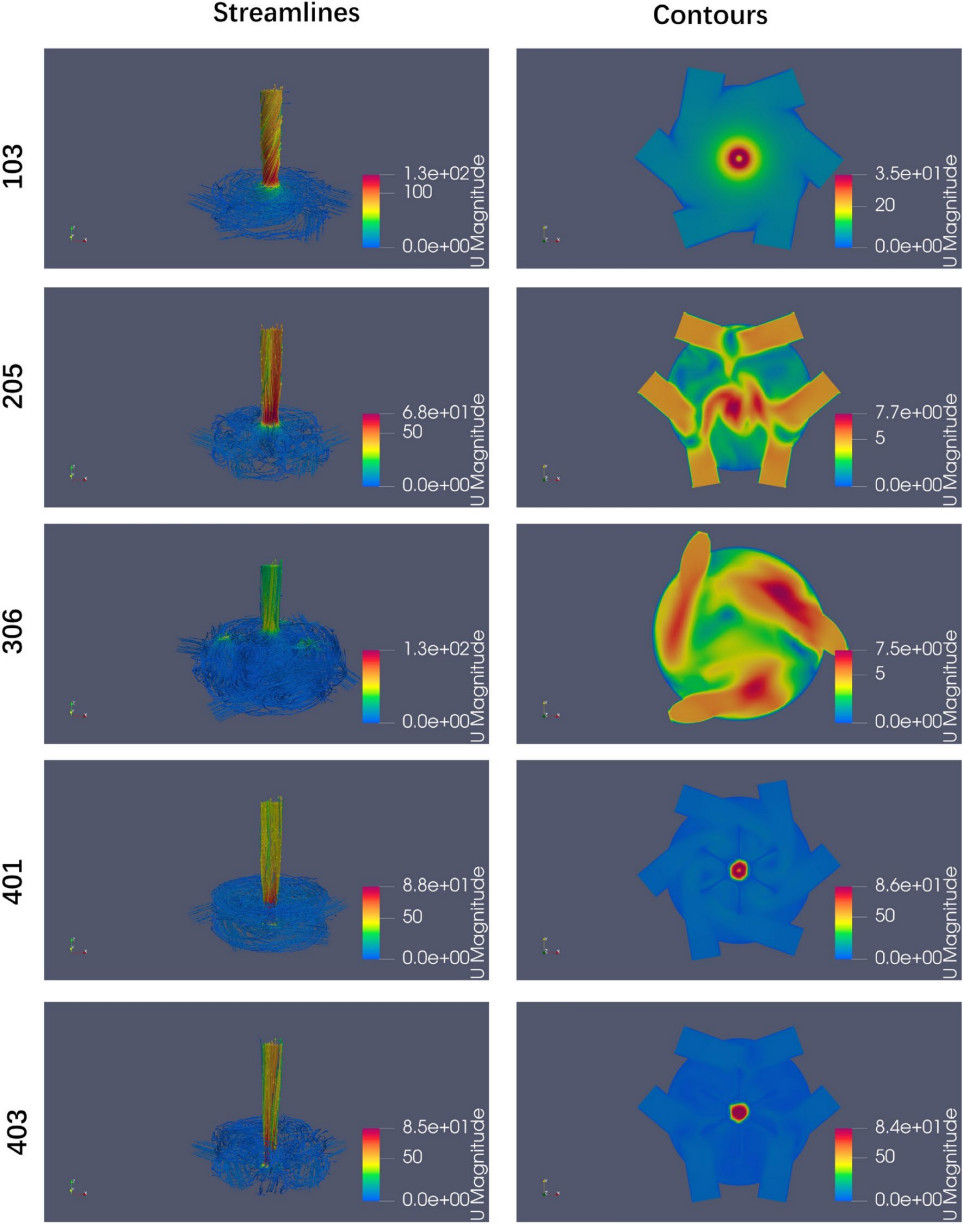
Streamlines and contours for selected Scheme 1.

From the perspective of fluid flow, in Case 1, water enters the vessel, rotates, and flows along the interior wall of the vessel. It then enters the center region and increases its speed of rotation before exiting. This rotating flow field is analogous to the centrifugal laminar flow formed by stirring. The size of the outlet pipe remains unchanged, whereas the diameter of the inlet pipe increases which results in increase in the outlet flow rate, internal pressure and fluid Reynolds number inside vessel. Ultimately, this leads to an increase in the value of the *Pe* number, as illustrated in Figs. 15(103) and 16(105).

In Case 2, the inlet pipes are arranged differently from those in Case 1. Once water enters the vessel, two strands of fluid collide with each other, resulting in the formation of forced turbulence. Figs. 15(205) and 16(201) provide detailed illustrations of this phenomenon. When combined with the flow field and value of *I*, as shown in Figs. 15 (306) and 16(304)(305), it can be concluded that an increase in the volume of the vessel enhances the forced turbulent flow within vessel. The flow fields depicted in Figs. 15(401)(403) and 16(404) illustrate that the addition of internal piping and baffles within the vessel affects the flow field both in cases 1 and 2.

### TOPSIS analysis and verification

The trend assumption, which shows that the trend of a single control parameter is not influenced by the number of meshes, is verified in section “Assumption verification”. However, for flow channel performance, a comprehensive multiparameter evaluation is necessary. Consequently, this section employs the TOPSIS method to rank schemes and ascertain the impact of the number of meshes.

The evaluation principle defines that larger*Pe* and *I* and smaller $\Delta P$ values are preferable. TOPSIS is employed for ranking. As previously discussed, the trends of *Pe* and *I* are negatively correlated. Consequently, distinct setting methods are employed for the *Pe* and *I* weight values, designated strategies 1 and 2, respectively. These correspond to cases 1 and 2 in the previous section. The rubric utilized is $[Pe, I, \frac{1}{\Delta P} ]$, and the weights assigned to strategy 1 are [0.8, 0.1, 0.1], whereas those assigned to strategy 2 are [0.1, 0.8, 0.1]. To calculate the ranking of the five groups of schemes, the TOPSIS method is applied to mesh series ${F-25, F-20, F-15, F-10, F-5}$.

The calculation steps for series ${F-5}$ are employed as an example to demonstrate the specific calculation process. The data column ${F-5-Pe, F-5-I}$ and ${F-5-\Delta P}$ can be extracted from the data presented in Fig. 11. The desired scheme requires larger *I* and *Pe* values and smaller $\Delta P$. The differential pressure is judged in the opposite way to the other two terms. It is necessary to unify the judgement method first, and after taking the inverse of each term in the data column ${F-5-\Delta P}$ to to obtain data column ${F-5-\frac{1}{\Delta P}}$, the judgement method changes to the larger the better. Then it can be combined to form the initial data matrix ${O = [F-5-Pe, F-5-I, F-5-\frac{1}{\Delta P}]}$. Subsequently, the normalised performance matrix *Z* is calculated using Eq. 2, with the selected weights [0.8, 0.1, 0.1] or [0.1, 0.8, 0.1] brought into Eq. 3 to obtain the weighted matrix *V*. Finally, the positive ideal scheme set and the negative ideal scheme set of the matrix are determined using Eqs. 4 and 5. According to Eqs. 6 and 7, the distance between each scheme and the positive ideal scheme and the negative ideal scheme is calculated. This is then used to calculate the score of the scheme using Eq. 8. Finally, the schemes can be ranked according to these scores.

The results shown in Fig. 13a, b illustrate five-group ranking plots of two strategies, whereas Fig. 14a, b depict curves generated by extracting the maximum and minimum rankings of each scheme within five sets of data.

Scheme 102 represents initial one and serves as the basis for evaluation, as indicated by the dotted line in Fig. 14**a** and b. When the maximum rankings of the other schemes are lower than the dotted line, the evaluation of the schemes is not influenced by the number of meshes. Schemes 103, 105, 401 and 402 are selected from Fig. 14a, and accordingly, Schemes 103, 105, 201, 205, 304, 305, 306 and 404 are selected from Fig. 14b.

**Figure 16:**
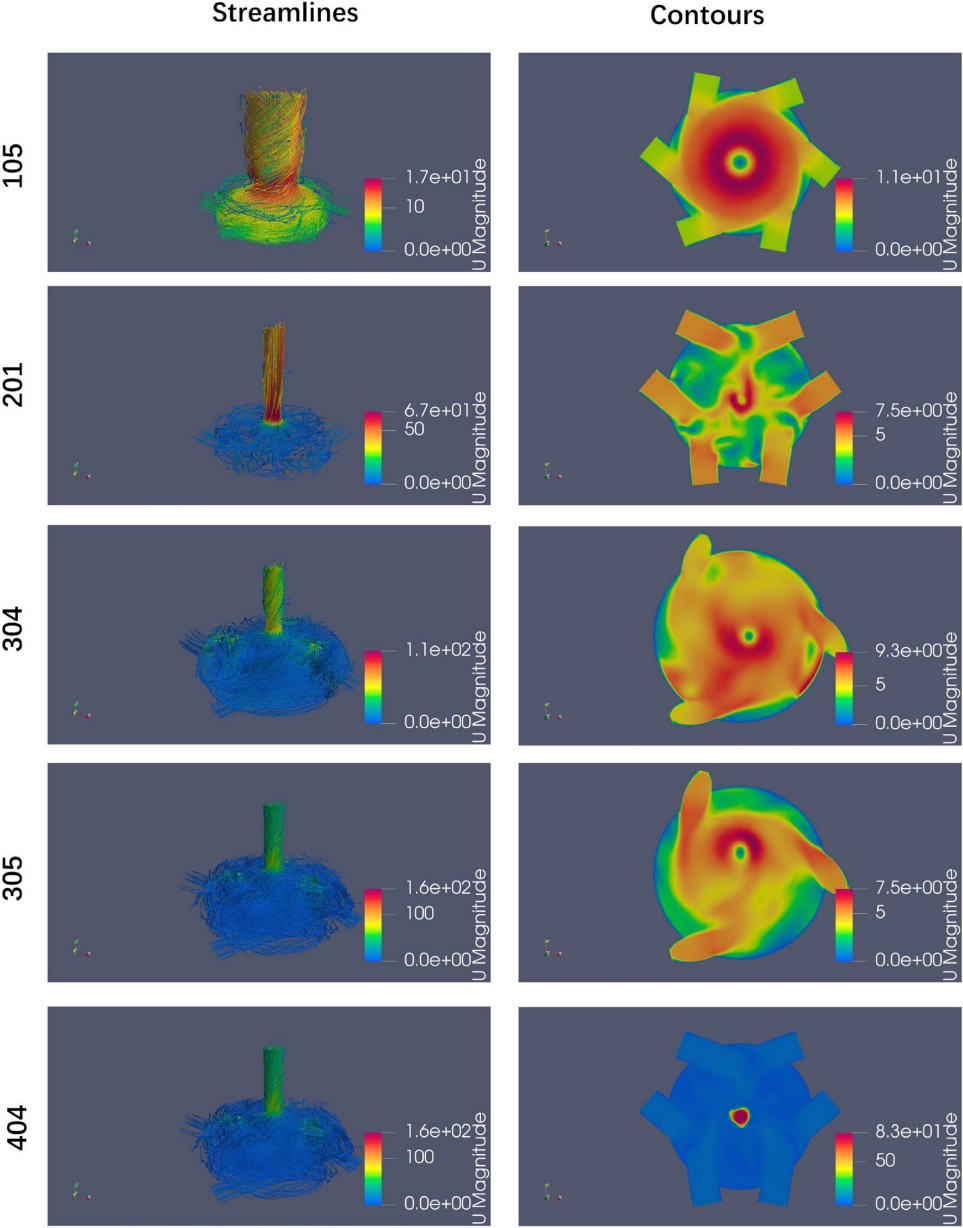
Streamlines and contours for selected Scheme 2.

Strategies 1 and 2 are the foundations for discussing the selected schemes. Schemes 103 and 105 are selected for both strategies. For Scheme 103, the *Pe* and $\Delta P$ values are the highest, and the *I* value is the lowest, which shows that the flow rate increases with constant inlet velocity when the inlet piping diameter increases, see Fig. 11b–d. However the diameter of the outlet is unchanged, which results in an increasing velocity at the outlet and in the vessel, and more potential energy is needed. For Scheme 105, the extremely low $\Delta P$ value results in a high ranking, as shown in Figs. 11b and 16(105). Therefore, one design direction may be considered to increase the flow rate at high pressure.

Schemes 401 and 402 under strategy 1 demonstrate that the internal parts equipped can increase the *Pe* value. Both their $\Delta P$ and *Pe* values are higher than those of the initial scheme which results in ranking. A comparison of the rankings of 401 and 402 reveals that the two rankings are equal, indicating that baffles are of minimal use and that only internal piping needs to be considered, as shown in Figs. 11b, c and 8.

Strategy 2 is very different from strategy 1; that is, Scheme 205 can replace scheme 102 as the basis for evaluation in strategy 2. Thus only Schemes 305 and 306 should be considered as better design directions, which expand the internal space of the vessel and modify the relatively opposite spatial arrangement of two pipes in plane to a spatial relative opposite arrangement. Parameter $\alpha 2$ is very effective for the above optimization, and parameter *Exd* is studied more effectively, as shown in Fig. 8. Furthermore optimization failure of Schemes 301, 302, 303 and 304, as shown in Fig. 14b, indicates that this direction still needs to be studied too. In addition, the direction of the internal parts is considered to lack research value on the new evaluation basis.

The minimum ranking of Scheme 102 is a crucial factor in the evaluation process. According to Fig. 13a, Scheme 102’s ranking is the same and lowest at F-20, F-15, and F-10, and the same and highest at F-25 and F-5. The lowest ranking of Scheme 102 in Fig. 13b is contributed by F-25. In this instance, the number of meshes does not affect the results of the design scheme simulation system.

### Trend sensitivity

As illustrated in Fig. 12a, as the number of meshes increases and the mesh size decreases, there is a clear detrending of the *Pe* trend curve, indicating that the data of each scheme converge. The length scales in Eq. 13 become linearly related to the cell-to-cell distance. Consequently, when the mesh size is sufficiently small, length scale continues to decrease, causing the *Pe* value to converge and the sensitivity to be lost.

## Conclusion

The results of the verification in section “Case study” indicate that the simulation system for DSTs is beneficial for expanding the use of CFD to develop design ideas and improve design efficiency in the process of comparing the control parameters of multiple schemes.

The simulation system is based on a fully automated process, which allows for the efficient production of data with physical meaning. These data can be stored by the requisition and can also be used for subsequent data analysis. This paper aims to overcome the limitations of traditional simulation processes by introducing a new approach based on templated geometrical modeling. This approach enables the direct generation of mesh files via the JIACFD toolset, which can be combined with other mature flow field computation software and postprocessing software to establish a fully automated simulation process that has been verified to be feasible.

The verification of the trend assumption of the control parameters proposed in section “Control parameter trend assumption (CPTA)” and the application of TOPSIS for multiparameter analysis to derive ranking results demonstrate that the application of trend analysis can be effective in obtaining comparative results when the accuracy of simulated data is not high. Consequently, the whole system can reduce the simulation requirements, number of meshes and simulation time. The use of structural hexahedral meshes in this paper establishes that the preprocessing time is controllable and efficient. Consequently, the system exhibits acceptable efficiency.

The results of the analysis in section “Trend correlation of different control parameters” indicate that independent control parameters are correlated under trend analysis. Furthermore, the flow field mechanism of different design schemes can be explored by combining DSTs that conform to engineering logic with control parameter trends. This approach is conducive to innovative design and design optimization needs, which also facilitates the expansion of design ideas and the identification of better design directions. Consequently, this system is applicable in engineering design and analysis.

During the analysis process, as the number of meshes increases, the curve of *Pe* gradually decreases. Consequently, this paper shows that trend analysis is sensitive, with a steeper curve indicating greater sensitivity and an otherwise flatter curve indicating reduced sensitivity. The trend sensitivity test can be used to determine whether relevant parameters or calculation models are suitable for simulation analysis.

## Future work

Further verification of the CPTA is needed with respect to cases of unstructured meshes and the presence of more branches in DSTs. The simulation trend curve should be independent of the mesh type. After improving the hardware performance, more different types of cases can be employed for simulation to verify the generality of the system. Furthermore, the incorporation of a variety of mesh series is beneficial in evaluating the CPTA.

A comprehensive investigation of trend sensitivity is necessary to identify the factors influencing this phenomenon. Additionally, preliminary simulations have indicated that the flow field simulation solver and turbulence model may influence trend sensitivity. If some methods are capable of accurately simulating a single scheme while the control parameters exhibit minimal trend sensitivity in the DST system, its overall usability may be questionable. This implies that the automated system can invoke the LES and DNS models as well as other RANS models for simulations, providing insights into the relationship between turbulence models and the changing scales of the flow channel.

Additional geometry modules must be developed to facilitate the generation of more design schemes. These modules must be capable of combining different designs to achieve efficiency and speed in the generation of design schemes. The fundamental principle the guiding development of these modules is that they are capable of satisfying a sufficiently large number of geometric variables, ultimately leading to the generation of an effective flow channel structure on the basis of random variables. Therefore, based on a fully parametric geometric modelling approach, the application of machine learning techniques is explored to accelerate the establishment of novel flow channel structure templates.

A two-level parallel simulation platform can be constructed to increase the operational efficiency of the system. Initially, we propose 5-10 engineer stations equipped with high-core CPUs, installation of independent and identical systems and necessary software, and subsequent establishment of a small local management system to assign and unify simulation tasks and simulation results of design schemes. As the scale of computation increases, it is considered that a local area network (LAN) be formed with the incorporation of a local area management program for the administration of the first level. Furthermore, the implementation of the two-level computation server is a more advanced option.

## Data Availability

The data that support the findings of this study are openly available at http://www.jiacfd.com/#/download/download_mixer.
